# Regulation of human microglial gene expression and function via RNAase-H active antisense oligonucleotides in vivo in Alzheimer’s disease

**DOI:** 10.1186/s13024-024-00725-9

**Published:** 2024-04-24

**Authors:** Lina Vandermeulen, Ivana Geric, Laura Fumagalli, Mohamed Kreir, Ashley Lu, Annelies Nonneman, Jessie Premereur, Leen Wolfs, Rafaela Policarpo, Nicola Fattorelli, An De Bondt, Ilse Van Den Wyngaert, Bob Asselbergh, Mark Fiers, Bart De Strooper, Constantin d’Ydewalle, Renzo Mancuso

**Affiliations:** 1 Neuroscience Discovery, Janssen Research & Development, Janssen Pharmaceutica NV, 2340 Beerse, Belgium; 2 VIB-KU Leuven Center for Brain & Disease Research, Leuven, 3000 Belgium; 3 Laboratory for the Research of Neurodegenerative Diseases, Department of Neurosciences, Leuven Brain Institute (LBI), KU Leuven, Leuven, 3000 Belgium; 4 Preclinical Development & Safety, Janssen Research & Development, Janssen Pharmaceutica NV, 2340 Beerse, Belgium; 5 Discovery Sciences, Janssen Research & Development, Janssen Pharmaceutica NV, 2340 Beerse, Belgium; 6 UK Dementia Research Institute, University College London, London, W1T 7NF UK; 7 MIND Lab, VIB Center for Molecular Neurology, VIB, 2610 Antwerp, Belgium; 8 Department of Biomedical Sciences, University of Antwerp, 2610 Antwerp, Belgium; 9 Neuromics Support Facility, VIB Center for Molecular Neurology, University of Antwerp, 2610 Antwerp, Belgium; 10 Neuromics Support Facility, Department of Biomedical Sciences, University of Antwerp, 2610 Antwerp, Belgium

**Keywords:** Microglia, Neuroinflammation, Alzheimer’s disease, Antisense oligonucleotide, TREM2, APOE

## Abstract

**Background:**

Microglia play important roles in maintaining brain homeostasis and neurodegeneration. The discovery of genetic variants in genes predominately or exclusively expressed in myeloid cells, such as Apolipoprotein E (*APOE*) and triggering receptor expressed on myeloid cells 2 (*TREM2*), as the strongest risk factors for Alzheimer’s disease (AD) highlights the importance of microglial biology in the brain. The sequence, structure and function of several microglial proteins are poorly conserved across species, which has hampered the development of strategies aiming to modulate the expression of specific microglial genes. One way to target *APOE* and *TREM2* is to modulate their expression using antisense oligonucleotides (ASOs).

**Methods:**

In this study, we identified, produced, and tested novel, selective and potent ASOs for human *APOE* and *TREM2*. We used a combination of in vitro iPSC-microglia models, as well as microglial xenotransplanted mice to provide proof of activity in human microglial in vivo.

**Results:**

We proved their efficacy in human iPSC microglia in vitro, as well as their pharmacological activity in vivo in a xenografted microglia model. We demonstrate ASOs targeting human microglia can modify their transcriptional profile and their response to amyloid-β plaques in vivo in a model of AD.

**Conclusions:**

This study is the first proof-of-concept that human microglial can be modulated using ASOs in a dose-dependent manner to manipulate microglia phenotypes and response to neurodegeneration in vivo*.*

**Supplementary Information:**

The online version contains supplementary material available at 10.1186/s13024-024-00725-9.

## Background

Microglia are the main resident immune cells in the central nervous system (CNS). They have a major role in supporting CNS homeostasis in various ways [1]. They contribute to neurogenesis, synaptogenesis and synaptic plasticity [2, 3]. They also provide trophic support to neurons, and they regulate immune cell recruitment in response to exogenous or endogenous damaging stimuli [4, 5]. The identification of disease-associated and disease-causing genetic variants in genes expressed predominantly or exclusively by myeloid cells indicates that microglia have a central contribution to disease pathogenesis [1, 6–10]. Approximately half of the Alzheimer’s disease (AD) risk loci identified by genome-wide association studies and meta-analyses are implicated directly in microglial biology and neuroinflammation [6, 11]. These include two of the strongest risk genes that encode for Apolipoprotein E (*APOE*) and Triggering receptor expressed in myeloid cells-2 (*TREM2*) [12]. Microglia are also a central element in the pathogenesis of other diseases including frontotemporal lobal degeneration and amyotrophic lateral sclerosis, as they are highest expressors of progranulin (*GRN*) and *C9orf72* in the brain [13, 14]. Thus, modulating microglial function is an attractive avenue to tackle neurodegeneration.

An increasing amount of evidence from functional studies indicates that microglial responses play a significant role in neurodegeneration [15, 16]. Several studies using single-cell RNA sequencing in mouse systems demonstrated that homeostatic microglia express a unique set of genes and cell surface proteins that discriminate them from peripheral myeloid cells as well as from damage or disease-associated microglia (DAM) [17–21]. We have recently characterized the full spectrum of cell states induced in human microglia by amyloid-β pathology using a xenotransplantation model [22]. The modulation of these states presents an appealing strategy to modify neuroinflammation and ameliorate or arrest the progression of AD and other neurodegenerative disorders. Manipulating the expression of disease risk-associated microglial genes may be a new strategy to alter microglial phenotypes and define the role(s) of microglia in neurodegeneration.

One way to selectively alter the expression of specific genes is by using antisense oligonucleotides (ASOs). ASOs are short single-stranded chemically modified hybrid DNA molecules that bind to their cognate target RNA by complementary Watson–Crick base-pairing to modulate their target RNA [23, 24]. There are two types of ASOs: RNase-H active ASOs and steric blocking ASOs [25–28]. The endogenous RNase-H enzyme recognizes the RNA–DNA heteroduplex that are formed when an ASO binds to its target RNA and catalyzes the degradation of the RNA [25, 26]. Cleavage at the site of the ASO binding results in destruction of the target RNA, culminating in target gene expression silencing [25, 26]. In contrast, steric blocking ASOs bind to their target RNA but are designed so that they do not induce their degradation [27, 28]. Instead, they can mask specific sequences to interfere with RNA-RNA, RNA–protein and even RNA–DNA interactions [27–29]. The development of a wide range of chemical modifications of the bases and the sugar-phosphate backbone substantially improved the ASO’s binding affinity to serum proteins and target RNA, immunogenicity, and endonuclease resistance [30–34]. Local delivery into the cerebrospinal fluid (CSF) via lumbar puncture results in widespread distribution of ASOs throughout the brain and spinal cord with limited peripheral exposure. ASOs that are locally delivered into the CNS can exert their function for extended periods of time, lasting several weeks, and making them an attractive therapeutic modality for CNS disorders [35, 36]. The FDA approved nusinersen is an ASO that modifies an alternative splicing event in the *SMN2* pre-mRNA, and is used as a treatment for spinal muscular atrophy [37–39]. Moreover, several RNase-H active ASOs are currently in late-stage clinical trials for CNS indications [40–42].

Most CNS-targeting ASOs approved by the FDA or ASOs currently evaluated in clinical trials target neuronal genes, with the only exception of an ASO targeting GFAP for Alexander Disease [43]. This includes several ASOs that target the gene encoding Tau, a protein that forms large, insoluble highly phosphorylated aggregates called neurofibrillary tangles in brains of AD patients [44]. However, it is currently unclear if ASOs targeting disease risk genes in microglia can also be used therapeutically to alter the response of human microglia to pathology. It has been demonstrated that downregulating mutant human *APOE* expression with ASOs in the brains of transgenic AD mouse models reduces pathological hallmarks of their neurodegenerative phenotypes [45, 46]. However, *APOE* is expressed by various cell types in the CNS and it is unclear whether ASOs were able to reduce *APOE* expression specifically in microglia or if APOE secreted from other cell types in CNS contributed to the observed changes [45, 46]. Similarly, reducing mouse *Trem2* expression with ASOs in an AD mouse model ameliorates pathological hallmarks of disease [47]. However, there are pronounced species differences in sequence, structure, and function of microglial proteins, including *TREM2* [21, 48–52]. It is therefore not certain if reducing *TREM2* expression in human microglia would have the same beneficial effects.

Here, we explore the ASO-mediated RNase-H reduction of *APOE* and *TREM2* expression, two of the major risk genes for AD, in human microglia both in vitro and in vivo*.* We identify potent ASOs targeting human *APOE* and *TREM2* that reduce their target gene expression at the RNA and at the protein level in cultured human microglia. We demonstrate that *APOE*- and *TREM2*-targeting ASOs are pharmacologically active and can modify the phenotype of human microglia in vivo using a xenotransplantation mouse model grafted with human microglia [48, 53]. Finally, we assess the safety of our *APOE* and *TREM2* targeting ASOs. Our results show for the first time that ASOs targeting disease-associated risk genes are active in human microglia and can alter microglial cell states in vivo.

## Methods

### Antisense oligonucleotides

All ASOs were synthesized (Axolabs GmbH) as 20-mers with the 5 outer nucleosides on either end modified at the 2’ position of the ribose with methoxyethyl groups (2’MOE). The central 10 nucleosides were unmodified deoxynucleosides. All cytosines were methylated, and all internucleoside linkages were phosphorothioate. A total of 278 and 235 ASOs were synthesized to target *APOE* and *TREM2* pre-mRNA, including exonic and intronic regions, respectively. The lyophilized ASOs were reconstituted in phosphate-buffered saline (PBS) (Sigma-Aldrich, D8537), filtered through a 20 µm filter, and their final concentration was determined by measuring their absorbance at 260 nm. Multiple batches were synthesized. The lead ASO sequences are provided in Supplemental Table 1. To determine possible off-targets of the selected lead candidate ASOs, we used Basic Local Alignment Search Tool (blastn) analysis. Lead ASOs targeting *APOE* and *TREM2* did not overlap to any other human transcript (Suppl Table 1).

### Cultures of human cell lines

All immortalized cell lines were obtained from ATCC unless listed otherwise. THP-1 cells (TIB-202, CVCL_0006) were cultured in RPMI-1640 medium (Sigma-Aldrich, R0883) supplemented with 2 mM Glutamax (ThermoFisher, 35,050–038), 25 mM HEPES (ThermoFisher, 15,630–056), 10% (v/v) fetal bovine serum (FBS) (Biowest, S1810-500) and 50 µg/mL Gentamycin (ThermoFisher, 15,750–045). K-562 cells (CCL-243, CVCL_0004) were cultured in RPMI-1640 medium supplemented with 10% FBS, 2mM L-Glutamine (Sigma, G8540) and 50 µg/mL Gentamycin. SH-SY5Y cells (CRL-2266, CVCL_0019) were maintained in DMEM/F12 (Sigma, D9785) supplemented with 10% FBS, 0.1 mM NEAA (Gibco, 11,140,050) and 50 µg/mL Gentamycin. SK-N-MC cells (HTB-10, CVCL_0530) were maintained in MEM medium (Gibco, 41,090,028) supplemented with 10% (v/v) FBS, 1 mM Sodium Pyruvate (Gibco, 11,360,070), 17.9mM sodium bicarbonate (Sigma, S-5761), 0.1mM NEEA and 50 µg/mL Gentamycin. Kelly cells were obtained from DSMZ (ACC 355, CVCL_2092) and were cultured in RPMI-1640 medium with 10% (v/v) FBS, 2 mM L-Glutamine and 50 µg/mL Gentamycin. HCT-116 cells (CCL-247, CVCL_0291) were cultured in McCoy's 5A medium (Gibco, 16,600,082) with 10% (v/v) FBS, 100 U/ml Penicillin and 100 µg/ml Streptomycin (ThermoFisher, 15,140–122). Caco2 cells (HTB-37, CVCL_0025) were cultured in MEM medium supplemented with 20% (v/v) FBS, 0.1mM NEAA, 1 mM Sodium Pyruvate, 2 mM L-Glutamine and 50 µg/mL Gentamycin. SK-MEL1 cells were obtained from DSMZ (ACC 303, CVCL_0068) and cultured in DMEM (Gibco, 10,938,025) with 15% (v/v) FBS, 2 mM L-Glutamine and 50 µg/mL Gentamycin. T-46D cells (HTB-133, CVCL_0553) were maintained in RPMI-1640 medium with 10% (v/v) FBS, 2 mM L-Glutamine, 10 µg/mL insulin (Sigma, I9278), 100 U/ml Penicillin and 100 µg/ml Streptomycin. All cell-lines were split bi-weekly and were sub-plated in non-coated 96-well µclear plates (Greiner, 655,090) between 20,000–40,000 cells/well.

### Human iPSC-derived microglia (iMGL)

The iPSC lines used in the cellular assays were obtained from the EBiSC consortium (https://ebisc.org) (Suppl Table 2). UKBi011-A (APOE ε4/ε4; CVCL_LE34), UKBi011-A-3 (APOE ε3/3; CVCL_RX83), and UKBi011-A-1 (APOE KO/KO; CVCL_RM82) were used to assess the pharmacology of APOE ASOs in vitro. The human isogenic TREM2 iPSC lines BIONi010-C (parental; CVCL_1E68), BIONi010-C-7 (TREM2 R47H/R47H; CVCL_II86), and BIONi010-C-17 (TREM2 KO/KO; CVCL_RM88) were used to evaluate the activity of TREM2 ASOs in vitro. For off-target analysis using microarray the BIONi010-C (parental; CVCL_1E68) line was used. An additional wild-type iPSC line was included for ASO uptake validation (SIGi001-A; CVCL_EE38).

Human iPSC-derived microglia (iMGL) were differentiated using a method described by Cowley et al. [54, 55]. Briefly, iPSCs were dissociated with TrypLE select (ThermoFisher, 12,563,011) into single cell suspension and embryoid bodies (EBs) were produced in AggreWell800 (Stem Cell Technologies, 34,811) in mTeSR medium (Stemcell Technologgies, 85,850) supplemented with 50 ng/ml BMP-4 (Invitrogen, PHC9534), 50 ng/ml VEGF (PeproTech, 100–20) and 20 ng/ml SCF (Miltenyi, 130–096-693). The medium was refreshed daily by 75% for 3 consecutive days. Next, EBs were transferred to factory plates to create precursor factories by placing 10–20 EBs per well in X-VIVO 15 medium (Lonza, BE02-060F) containing 100 U/ml Penicillin and 100 µg/ml Streptomycin (ThermoFisher, 15,140–122), 2 mM Glutamax supplement, 0.05 mM 2-mercaptoethanol (ThermoFisher, 31,350–010), 0.1 µg/ml M-CSF (ThermoFisher, PHC9501) and 0.025 µg/ml IL-3 (ThermoFisher, PHC0031). The factories were cultured for 6–8 weeks (with weekly 50% medium change) before the cells were harvested for final differentiation into microglia. Microglia precursors were harvested for no more than once a week. At harvest, microglia precursors were plated at a density of 15,000 cells/well in non-coated 96-well µclear plates (Greiner, 655,090) and cultured in advanced DMEM/F12 medium (ThermoFisher, 12,634–010) containing 100 U/ml Penicillin and 100 µg/ml Streptomycin, 2 mM Glutamax supplement, 0.05 mM 2-mercaptoethanol, 10 ng/ml GM-CSF (ThermoFisher, PHC2015) and 100 ng/ml IL-34 (Peprotech, 200–34) for maximally 14 days (with 50% medium change every 2–3 days).

### RNA isolation and real-time quantitative PCR

Total cellular RNA was isolated using the RNeasy96 kit (Qiagen, 74,182) according to manufacturer’s protocol. Briefly, cells were lysed with RLT buffer at RT and an equal volume of 70% (v/v) ethanol was added. The mixture was pipetted 5 times and transferred to columns in which the RNA was bound to the filter by a vacuum system (Qiagen). The RNA was washed sequentially with the RW1 and RPE buffers provided in the kit. After the final RPE washing step, a centrifugation was applied to make sure any residual buffer was eliminated (6 min at 5,600 × g at room temperature (RT). The RNA was then eluted with RNase-free water by centrifugation at 5,600 × g at RT for 4 min. The RNA concentration was determined by spectroscopy using a Nanodrop (ThermoFisher, ND-8000).

Equal amounts of RNA were reverse transcribed using the high-capacity cDNA reverse transcription kit (ThermoFisher, 4,368,813) according to manufacturer’s instructions on a thermocycler using the following incubation steps: 10 min at 25 °C, 2 h at 37 °C, and 85 °C for 5 min.

Realtime-quantitative PCR (RT-qPCR) was performed on diluted cDNA mixed with PowerUp SYBR Green master mix (ThermoFisher, A25743) or SensiFast Sybr No Rox Mix (Meridan Bioscience, bio-98020) and primers at a final concentration of 500 nM. Exceptionally, human *MALAT1* and *SMN* levels were measured with a Taqman assay mixed with PrimeTime master mix (IDT DNA Technologies, 1,072,102). Primers amplifying regions of various human housekeeping genes were used to quantify and normalize the mRNA expression levels. We designed 4 different primer assays to quantify *APOE* and *TREM2* mRNA, each spanning an exon-exon boundary along the mature transcript (Suppl Fig. 1A,B, Suppl Table 3). All primers were purchased from IDT DNA Technologies are listed in Supplemental Table 3. Realtime detection of the mRNA quantities was done on a QuantStudio 12k Flex (ThermoFisher) or Quantstudio 7Pro (ThermoFisher) in 384 well plates using standard settings and with the following cycling parameters: 2 min at 50°C and 10 min at 95 °C, followed by 40 cycles of 15 s at 95 °C and 1 min at 60 °C. For SYBR green primers, a melt curve was included at the end of the run for 15 s at 95 °C, 1 min at 60 °C and 15 s at 95 °C. Alternatively, detection was done on a LightCycler 480 (Roche) with following parameters: initial denaturation (95°C, 10 min), 40 amplification cycles of denaturation (95°C, 10 s) followed by annealing and extension (60°C, 30 s), and a cooling step (40°C, 15 s).

RT-qPCR data were analyzed in qBase + (Biogazelle, version 3.2). First, at least 2 out of 8 most stable housekeeping genes were identified by a GeNorm analysis [56]. Next, target gene expression data were normalized to the geometric mean of the 2 most stable housekeeping genes and calibrated to a control condition. Expression data are expressed as calibrated normalized relative quantities (CNRQ) except when mentioned otherwise.

### Homogeneous Time-Resolved Fluorescence (HTRF) analysis of *APOE* and TREM2 protein levels

APOE and TREM2 levels were measured using an HTRF assay (Cisbio, 63ADK004PEH and 63ADK099PEH, respectively) following manufacturer’s instructions. Briefly, conditioned medium was collected prior to the ASO treatment and 48h-96h-7d post-treatment. The cells were lysed 7 days post treatment in 50 µl/well RIPA buffer (Sigma, R0278) supplemented with phosphatase inhibitors (PhosSTOP; Roche, 4,906,837,001) and protease inhibitors (Complete mini EDTA-free; Roche, 11,836,170,001) according to manufacturer’s instructions. Medium samples were diluted 1:4 for APOE and used undiluted for TREM2, where the cell lysates were diluted 1:2. Samples were incubated for 3 h at RT (APOE) or overnight at 4°C (TREM2) with a mixture of Europium Cryptate (donor) and d2 (acceptor) labelled antibodies following manufacturer’s instructions. Fluorescence was measured on an Envision (Perkin Elmer) at 665 nm and 615 nm (cycle 16,600 µs, delay 50 µs, # of flashes 50) and the HTRF ratio was calculated by multiplying the ratio of the signal at 665 nm over the signal at 615 nm by 10,000. The HTRF Δ ratio was calculated by the ratio of the samples minus the ratio of the background signal (unconditioned medium negative control). Fluorescence signal obtained from serially diluted recombinant APOE and TREM2 was used to interpolate the concentration of APOE and TREM2, respectively, and normalized for the dilution factor. The final data are expressed as ng/mL APOE and pg/mL TREM2, respectively.

### Fluorescence microscopy

For live-cell imaging using an IncuCyte Zoom (Essenbioscience/Satorius, software version 2016B), we treated cells with an Alexa Fluor-555 labelled *MALAT1* ASO and imaging was started approximately 5 min after addition of the ASOs using a 10 × or 20 × objective at 37 °C and 5% CO_2_. Images were obtained at frequent time points over time. Images/videos were used in a qualitative manner.

For live-cell imaging using an Opera Phenix High-Content Screening System (Perkin Elmer, HH14000000), cells were incubated with a CellMask™ Deep Red Plasma membrane dye (1:10.000, Thermofisher, C10046), LysoTracker™ Green dye (1:20.000, Thermofisher, L7526) and Hoechst 33,342 (1:10.000, Thermofisher, H3570). After 15 min the fluorescently labelled *MALAT1* ASO (custom made ASOs (IDT DNA) with Alexa 594 label on the 3 or 5 end) was added. Immediately after the addition of ASO, imaging was initiated using a 20 × or 40 × water immersion objective at 37 °C and 5% CO_2_. At least 11 fields per well with a Z-stack of at least 4 planes were imaged per condition. Images were taken every 20 min for the first 2 h, followed by image acquisition every 2 h for (until 24 h), and finally one image on day 2 and on day 3.

For immunocytochemical analysis, cells were fixed using 4% (w/v) paraformaldehyde (Pierce, 28,908) dissolved in PBS (Sigma, #D8537-500ML) for 15 min at RT. Cells were blocked and permeabilized with PBS supplemented with 0.1% (v/v) Triton X-100 (Sigma, 1,086,031,000) (PBS-T) and 5% (v/v) normal goat serum (Sigma Aldrich, G9023) for 1.5 h at RT. Washing steps were performed using PBS-T. Primary and secondary antibodies were diluted in PBS-T. A list of primary and secondary antibodies used can be found in Supplemental Table 4 and 5, respectively. Imaging was performed using an Opera Phenix High-Content Screening System (Perkin Elmer, HH14000000) using a 40 × water immersion objective, imaging at least 9 fields per well with a Z-stack consisting of at least 4 planes. If images were used in a quantitative manner, the analysis was performed using the Opera Phenix Harmony software (Perkin Elmer, version 4.9) using the maximum intensity projections. Fluorescence data were normalized to the number of nuclei.

### Hybridization ELISA

Cells and tissues were lysed in RIPA buffer (Sigma, R0278) supplemented with phosphatase (PhosSTOP; Roche, 4,906,837,001) and protease (Complete mini EDTA-free; Roche, 11,836,170,001) inhibitors. Cells were lysed at a ratio of 1 µl per 300 cells while tissues were lysed in 1 ml per 250 mg wet tissue weight. Tissue was homogenized in Lysing Matrix D (MP Biomedicals, 6913–500) using a FastPrep-24 5G (MP Biomedicals) with 3 cycles of 20 s at a speed of 6.0 m/s with 5 min between each cycle on ice to avoid sample heating. Lysates were then centrifuged for 5 min at 20,000 × g and 4 °C to remove debris.

Samples were hybridized with custom HPLC-purified probes (IDT DNA) consisting of unmodified 20-mer DNA sequence complementary to the ASO sequence. The probes contained a 5’ end digoxigenin label and a 3’ end tetraethylene glycol (TEG) spacer and biotin label. Each sample was hybridized with 2 nM probe diluted in hybridization buffer containing SSPE (Thermofisher, 15,591,043) and 0.4% (v/v) Tween-20 (Sigma, P7949). Hybridization was performed at 75 °C for 1 h. Serially diluted ASO with known amounts were used to define a standard curve.

The hybridized material was then spotted on neutravidin-coated plates (Thermofisher, 15,217) for 1h at RT. Plates were washed 2 times with Tris-buffered saline supplemented with 0.05% (v/v) Tween-20 (TBS-T, Sigma-Aldrich, T9039). Non-hybridized probe or any mismatched probe: ASO pair was destroyed by treating samples for 2 h with 300 U/ml S1 nuclease (Invitrogen, 18,001–016) diluted in S1 nuclease solution consisting of 300 mM sodium acetate, 10 mM Zn (CH_3_COO)_2_*2H_2_O, 50% (v/v) glycerol, pH 4.6. Each sample was then washed with TBS-T two times to remove non-hybridized material. Next, samples were incubated for 30 min at RT with an anti-digoxigenin antibody conjugated to alkaline phosphatase (Roche, 11,093,274,910) diluted in Super block buffer (Thermofisher, 37,536) containing 1 M sodium chloride (Promega, V4221). After 2 washes with TBS-T, ATToPhos substrate (Promega, S1000) was added to each sample and incubated at RT for 10 min. The reaction was stopped by adding 1 M sodium phosphate (J.T. Baker, 3824–01). After 15 min at RT, plates were read on an Envision (PerkinElmer) at an excitation of 423 nm and emission of 568 nm. Raw fluorescence data were exported and samples with unknown ASO levels were extrapolated based on the relative fluorescence units (RFU) and the known ASO concentration from the standard curve.

### Transcriptome analysis by microarray

RNA was extracted in a similar manner as described above. The RNA concentration was determined by spectroscopy using a Nanodrop (ThermoFisher, ND-8000). Amplification and labelling of total RNA were performed using the GeneChip® PICO Reagent Kit following the manufacturer’s protocol (ThermoFisher 2016, P/N 902790). Biotin-labeled target samples were hybridized to the Clariom™ GO Screen containing probes for 19,409 protein-coding genes. Target hybridization was processed on the GeneTitan® Instrument according to manufacturer’s instructions provided for Expression Array Plates (P/N 952361). Images were analyzed using the GeneChip® Command Console Software (GCC) (ThermoFisher). Microarray data were processed using the statistical computing R-program package limma (Version 3.42.0) and Bioconductor tools [57]. The gene expression values were normalized using Robust Multi-array Average (RMA) [58]. Individual probes were grouped into gene-specific probe sets based on Entrez Gene using the metadata package goscreenhuhsentrezg (version 25.0.0) [59].

### Electrophysiology

All experimental protocols were approved by the ethical committee of Janssen Pharmaceutica N.V. Primary neurons were freshly dissociated from embryonic E18-19 rat cortices as described previously [60] and plated onto 48-well MEA plates (Maestro Pro-167, Axion Biosystems). One day before plating the cells, each 48-well MEA plate was pre-coated with a polyethyleneimine (PEI) (0.1%) solution (Sigma, P3143), washed four times with sterile distilled water, and then allowed to dry overnight. On the day of plating, Laminin (20 μg/ml) (Sigma, L2020) was added to each 48-well plate which was then incubated for 1 h at 37 °C. Thereafter the neurons were cultured at 37 °C, 5% CO_2_ in Neurobasal medium (Thermofisher, 21,103–049) supplemented with 0.5 mM L-Glutamine (Thermofisher, 25,030,149) and 2% B27 (Thermofisher, 17,504,044).

Data analysis was performed using AxIs software (version 3.6.1.14, Axion Biosystems Inc.). The threshold for the spike detection was ≥ 5.2 × the standard deviation of the RMS (root mean square) noise. Statistical analysis consisted of expressing the treatment ratio of exposed wells (percentage change between the baseline and the treatment) normalized to the treatment ratio in control experiments. Normalized treatment ratios of *n* = *8* wells were averaged per condition. Each well of the MEA served as its control, and the changes in electrical activity elicited by the treatments were expressed as a percent of that control activity and normalized to the wells treated with the vehicle control PBS. The final concentration of PBS added to each well was 0.1% (1 μl/ml), which did not alter the pH or the ionic concentration of the medium.

### Human whole blood sampling

Human whole blood samples were collected into Sodium-Heparin BD Vacutainer of 4 healthy donors through voluntary blood donation approved by the Commission of Medical ethics of the ZNA according to European Union guidelines. The donors signed an informed consent and the collected samples were anonymized, and no further personal data was collected. The samples were diluted 1/10 in RPMI-1640 medium (Sigma-Aldrich, R0883) with 10% (v/v) FBS (Biowest, S1810-500), 100 U/ml Penicillin and 100 µg/ml Streptomycin (ThermoFisher, 15,140–122) and plated in non-coated 96-well µclear plates (Greiner, 655,090). The ASOs and positive controls (Toll-like receptor (TLR)-7 and TLR-8 agonists; CAS ID: 1,642,857–69-9 and 144,875–48-9) were added to the blood immediately after plating at 3 different concentrations (1, 2.5 and 10 µM). One day later, samples were collected by a centrifugation at 100 × g for 3 min. Supernatants were transferred to a 96-well PCR plate (ThermoFisher, AB0600), centrifuged at 2,500 × g for 2 min and transferred to a 96-well ½ Area OptiPlate (Perkin Elmer, 6,002,290) for storage at -80 °C until further use.

### Cytokine and chemokine profiling

Levels of interleukins (IL) IL-1β, IL-6, IL-8, IL-10, IL-12p70, interferon gamma (IFN-γ) and tumor necrosis factor alpha (TNF-α) in culture medium and human whole blood were determined simultaneously using a commercially available quantitative electrochemiluminescence assay (Meso Scale Diagnostics, K15008B-2). A commercially available GAPDH electrochemiluminescent assay (Meso Scale Diagnostics, K151PWD-2) was used to quantify GAPDH protein levels in cell lysates as loading control. The assays were performed following manufacturer’s recommendations. Briefly, medium or blood samples were diluted 1:2 in unconditioned medium or dilution buffer respectively and cell lysates were diluted 1:10. MSD plates were blocked with blocking buffer (MSD kit) for 1 h at RT and washed 3 × with washing buffer (PBS + 0.05% Tween-20). For the 7-plex cytokine assay a serially diluted calibrator was used to interpolate unknown values. Samples and calibrators were added and incubated for 2 h at RT after the detection antibody was added and incubated for 2 h at RT. The plates were washed 3 × with washing buffer and 2X read buffer T was added. For the GAPDH assay, samples were added and incubated for 1 h at RT. The plates were washed 3 × with washing buffer and the detection antibody was added for 1 h at RT. After washing 3 × with washing buffer 1 × read buffer T was added. Immediately after the read buffer was added, plates were measured using an MSD plate reader (Meso Scale Diagnostics, Sector Imager 6000). iMGL treated with Lipopolysaccharide (LPS) (Thermofisher, 00–4976-03) at 100 ng/mL (w/v) or whole blood treated with TLR-7 and TLR-8 agonists at 10 µM were used as a positive control to induce pro-inflammatory cytokine release. Results were analyzed using MSD DISCOVERY WORKBENCH software (Meso Scale Diagnostics, version 4.0.12). For the cytokine release analysis, the unknown samples were interpolated to the standard curve. GAPDH values were normalized to the untreated samples and further used as a normalization factor for the cytokine release measures.

### Transgenic mice

Homozygous *App*^*NL−G−F*^[61] mice on the C57BL/6J background [62] were crossed with *Rag2*^*−/−*^* IL2rγ*^*−/−*^* hCSF1*^*KI*^ mice purchased from The Jackson Laboratory (strain #017708) to generate *App*^*NL−G−F*^ mice on a background allowing transplantation and survival of human microglia [48, 53, 63]. Mice were bred and housed in local facilities at KU Leuven under a 14h light and 10h night cycle with food and water ad libitum. All experiments were approved by Ethical Committee of Laboratory Animals of the KU Leuven according to local (ECD project number P177/2017) and European Union guidelines.

#### Xenotransplantation of human microglia

Generation of microglia from human embryonic stem cells (hESC) was based on the previously described MIGRATE protocol [53]. First, the hESC H9 cell line (WA09 WiCell Research Institute, CVCL 9773) was seeded and induced towards EB formation, by using mTeSR1 media (Stemcell Technologies, 85850) supplemented with 50ng/ml BMP4 (PeproTech, 125–05), 50ng/ml VEGF (PeproTech, 100–20) and 20ng/ml SCF (PeproTech 300–07). Next, the generation of hematopoietic cells was initiated by culturing EBs in X-VIVO 15 media supplemented with 50 ng/ml SCF (PeproTech 300–07), 50 ng/ml M-CSF (PeproTech, 300–25), 50 ng/ml IL-3 (PeproTech, 200–03), 50 ng/ml Flt3-ligand (PeproTech, 300–19) and 5 ng/ml TPO (PeproTech, 300–18). Lastly, EBs were cultured in X-VIVO 15 media supplemented with 50 ng/ml Flt-3-ligand (PeproTech, 300–19), 50 ng/ml M-CSF (PeproTech, 300–25) and 25 ng/ml GM-CSF (PeproTech, 300–03) for the induction of the final myeloid lineage producing progenitor cells.

For in vitro experiments, progenitor cells were collected on day 25 and differentiated into microglia in differentiation medium described in Abud et al [64] (TIC; DMEM/F12, glutamine (2 mM) (ThermoFisher, 10,565,018), N-acetylcysteine (5 μg/ml) (Sigma-Aldrich, A7250), insulin (1:2 000) (Sigma-Aldrich, I9278), Apo-Transferrin (100 μg/ml) (Sigma-Aldrich, T1147), sodium selenite (100 ng/ml) (Sigma-Aldrich, S9133), cholesterol (1.5 μg/ml) (Sigma-Aldrich, C4951) and heparan sulfate (1 μg/ml) (Ams Bio, GAG-HS01) supplemented with 50 ng/ml IL-34 (PeproTech, 200–34), 50 ng/ml M-CSF (PeproTech, 300–25), 10 ng/ml CX3CL1 (Peprotech, 300–31) and 25 ng/ml TGF-β (PeproTech, 100–21)) for 7 days, with half of the media changed every other day. Cells were cultured in a humidified incubator at 37 °C and 5% CO_2_.

For xenotransplantation experiments, progenitor cells were harvested on day 18. Prior to transplantation, newborn mice were intraperitoneally injected with CSF1R inhibitor BLZ945 (Seleck, S7725) at a dose of 200 mg/kg body weight for 2 consecutive days to deplete endogenous mouse microglia. Subsequently, progenitor cells were collected at a final concentration of 250,000 cells/µl in PBS. Four days old pups were anesthetized by hypothermia and injected bilaterally with 1 µl of cell suspension using the following coordinates from bregma: anteroposterior, − 1 mm; mediolateral, ± 1 mm. Mice were allowed to recover on a heating pad at 37°C and were then transferred back to their cage.

### Intracerebroventricular delivery of ASOs

ASO stock solution (50 mg/ml) was diluted in PBS under sterile conditions and administered via intracerebroventricular (icv) injection as previously described [65]. Briefly, mice were anesthetized by continuous isoflurane inhalation and injected bilaterally with 2.5 µl of vehicle (PBS) or ASO (dose ranging from 3 to 125 µg) using the following coordinates from bregma: anteroposterior, − 0.22 mm; mediolateral, ± 1 mm; dorsoventral, -2.75 mm. The injection was done over 2 min and the needle was held in place for 5 min after the infusion. Mice were allowed to recover on a heating pad at 37°C and were then transferred back to the cage. Mice were monitored daily and were sacrificed 1 or 4 weeks after the injection.

### Tissue harvest and isolation of human and mouse microglia from mouse brain

Animals were sacrificed by overdose with sodium pentobarbital (Dolethal, Vetoquinal) and transcardially perfused with heparinized ice-cold PBS (5 U.I./ml heparin (LEO, 030710) in PBS). Liver, kidney, and spinal cord were immediately collected and snap frozen in liquid nitrogen. After removing cerebellum and brainstem, brains were dissected into three pieces; one was snap frozen in liquid nitrogen for bio-analysis and HTRF assays, a second one was fixed in 4% PFA (Sigma Aldrich, 1.00496.5000) for immunohistochemistry (IHC), and the third one was collected in FACS buffer (PBS, 2% FBS (Life Technologies, 10,270,106), 2 mM EDTA (Sigma-Aldrich, E7889) for further tissue homogenization and microglia isolation.

For the isolation of human and mouse microglia, the brains were collected as described above and were enzymatically dissociated using the Neuronal Dissociation Kit (P) (Miltenyi, 130–092-628) following manufacturer’s instructions. Actinomycin D (Sigma-Aldrich, A1410) was added to solutions used from tissue collection to myelin removal step, at a final concentration of 5uM. Dissociated samples were filtered through a cell strainer (70-µm mesh) and centrifuged at 300g for 15 min at 4°C. Pellets were resuspended in a 30% Percoll solution (Merck, 17–5445-02) in FACS buffer and centrifuged at 300 × g for 15 min at 4 °C. Myelin layer and debri were carefully removed and the supernatant was discarded. Then, the cell pellet enriched in microglia was washed with FACS buffer and incubated with FcR blocking solution (Miltenyi, 130–092-575) according to manufacturer’s instructions. After the blocking step, cells were washed with FACS buffer and incubated with the following antibodies for 30 min at 4°C: anti-CD11b (1:50, Milteny, 130–113-806), anti-hCD45 (1:50, BD Bioscience, 555485), anti-mCD45 (1:200, BD Bioscience, 563890). E780 (1:1000, Thermo Fisher, 65–0865-14) was used as viability marker. Human and mouse microglia were sorted as CD11b + hCD45 + and CD11b + mCD45 + cells, respectively, using the Miltenyi MACS Quant Tyto cell sorter (Suppl Fig. 2). Sorted cells were pelleted and stored at -80°C.

### Reanalysis of Mancuso et al. data

Data from Mancuso et *al.*, 2022 [22] was used to compare the transcriptomic impact of the ASO treatments to the profile of *TREM2* and *APOE*-KO human microglia in vivo. Single-cell data of xenografted microglia in 6-month-old *App*^*hu/hu*^ and *App*^*NL−G−F*^ mice were extracted from the dataset. The iPSC lines used were: UKBi011-A-3 (APOE ε3/3; CVCL_RX83), UKBi011-A-1 (APOE KO/KO; CVCL_RM82), BIONi010-C (APOE ε3/KO; CVCL_1E68) and BIONi010-C-3 (APOE KO/KO; CVCL_II82) from the EBiSC consortium (https://ebisc.org), and hESC H9 (WA09 WiCell Research Institute, CVCL 9773) and H9-TREM2 KO [66]. Mice engrafted with hESC H9 wild-type-derived cells were used to produce Suppl Fig. 10A-B. Mice engrafted with TREM2-KO or APOE-KO cells (and respective isogenic controls) were used for differential expression analysis to produce Fig. 5. Cell states annotations and marker genes were assigned according to the original manuscript. Differentially expressed genes were found by applying the *FindMarkers()* function from the *Seurat R* package for side-by-side comparisons of transplanted cell lines. The comparisons were performed with the following parameters: *assay* = *"SCT", test.use* = *"wilcox", min.pct* = *0, logfc.threshold* = *0*. We used the Wilcoxon rank sum test to calculate P-values. We performed DE on the "SCT" assay calculated from *SCTransform()*, since Pearson residuals resulting from regularized negative binomial regression effectively mitigate depth-dependent differences in differential expression, as described by [67]. We tested all genes without applying thresholds on average fold-change between conditions or minimum fraction of cells expressing the genes. Only genes with their adjusted P value < 0.05 (post-hoc, Bonferroni correction) were considered as significant.

### RNA seq data analysis

Total RNA extraction from sorted human microglia followed a methodology similar to the one detailed earlier. The study encompassed six distinct experimental groups. For each treatment and vehicle group, sorted human microglia were gathered from four animals. Consequently, the RNAseq analysis involved a total of 24 animals distributed across six experimental categories: 1-week treatment of APOE ASO-1, 4-week treatment of APOE ASO-1, 1-week treatment of TREM2 ASO-171, 4-week treatment of TREM2 ASO-171, and the respective vehicle groups for both 1 week and 4 weeks. RNA samples were then sent to BGI (bgi.com) for library preparation and sequencing. Samples were sequenced on their DNBSEQ-G400 platform. Sequencing data were mapped using STAR version = 2.7.10 against the joined reference library by combining the *Mus musculus* reference genome (mm10/GRCm38) and the human genome (GRCh38). The full count matrix was produced by FeatureCount v1.6.3 from the Subread package [68], using reads mapped to the human genome. We conducted differential expression analysis comparing the treatment and vehicle groups using the DEseq package [69], which streamlines standard differential expression analysis procedures in a single function *DEseq*. In this analysis, a design matrix was constructed for each treatment group, with the corresponding duration-matched vehicle group as the reference group. We then employed the Wald test to ascertain the log2fold changes and associated p-values for the comparison. To control for multiple testing, we applied the false discovery rate (FDR) adjustment using DEseq2's default Benjamini–Hochberg procedure.

### Microglial subtype gene set enrichment analysis

Subtype-signature genes are selected from the top 50 most significantly upregulated genes (adjusted p-value < 0.05) compared to other sub-types by Mancuso et al. 2022 [22]. Enrichment scores are calculated by using the Gene Set Enrichment Analysis Preranked module [69] for these gene sets and tested among all genes detected in isolated microglial cells, sorted from up to downregulated genes based on the log-fold changes calculated from differential expression analysis between treatment and vehicle groups (described in previous section and Suppl Fig. 12A-D). Significant GSEA enrichment was denoted by FWER < 0.05.

### Histological analysis

Brains were fixed in 4% PFA solution overnight. Serial coronal sections (35 um thick) were cut with a vibrating microtome and collected in free-floating conditions. Sections were kept at -20°C in cryoprotectant solution (PBS supplemented with 30% ethylene glycol (MP, 151,089) and 30% glycerol (Sigma, G5516)). For IHC, sections were permeabilized with 0.2% Triton X-100 (Sigma-Aldrich, X100) in PBS for 15min, and subsequently blocked for 1h with 2% normal donkey serum in PBS-0.2% Tween 20 (VWR, 0777). After washes with PBS-0.1% Tween 20, sections were incubated with primary antibodies at 4°C overnight. After washes with PBS-0.1% Tween 20, sections were incubated for 1h at room temperature with appropriate secondary antibodies. For the IHC with hCD9, hP2RY12, Iba1, and X-34 in Fig. 4 and Supplemental Fig. 11, the protocol was modified as follows: sections were permeabilized with 0.1% Tween 20 (Sigma-Aldrich, P1379) in PBS for 15min at room temperature and blocked with 5% normal donkey serum in PBS-0.1% Tween 20 for 1h at room temperature. Washing steps were performed PBS 0.1% Tween 20. A list of primary and secondary antibodies used can be found in Supplemental Table 4 and 5. DAPI (ThermoFisher, 62,248) was used to stain nuclei, and sections were mounted with Mowiol–DABCO (Sigma-Aldrich, 81,381) mixture or FluorSave (Calbiochem, 345,789). X-34 was used to stain amyloid plaques. Briefly, after permeabilization and before blocking step, sections were incubated for 20 min at room temperature with X-34 (1:1000, Sigma-Aldrich, SML1954) in 40% (v/v) ethanol solution supplemented with 20 mM NaOH and then washed with 40% (v/v) ethanol in PBS. Images were acquired at 20 × or 60 × magnification using Nikon A1R Eclipse confocal system or Nikon Ti2 Widefield microscope. Distribution of ASO in the brain was analyzed using Fiji Image J software. Threshold was set using brain sections from vehicle-treated mice. After setting the threshold, integrated density was measured and normalized to surface area. To measure microglial activation at the site of Aβ plaques in relation to the rest of the tissue, the center of the X-34 labeled plaques were selected and signal intensities of microglial markers (hCD9, hP2RY12) were extracted at rings (annuli) of incrementing diameter surrounding the plaque center. The measurements were executed via a macro in Fiji software. Only plaques surrounded by human cells (i.e., hP2RY12 positive cells) were considered for the analysis. The acquisition of the images and the analysis was carried out blinded to avoid introducing unconscious bias. To quantify Aβ plaques, 3 sections per mouse were acquired at 4 × magnification on Nikon A1R Eclipse confocal system. Plaques were identified as X-34 positive structures; and plaque number, size and volume were analysed using NIS software. Plaque number and volume was recorded for the whole brain section, plaque size was expressed as average size of all plaques measured in one section.

### RNAscope

RNAscope analysis was performed according to the instructions by the manufacturer on the Manual Fluorescent Multiplex kit v2 (ACDbio, 323,100) on PFA fixed tissue sections. Sections were air dried and incubated with Protease IV for tissue digestion and then incubated with the following probes for 2h at 40°C: Hs-APOE-C3 (ACDbio, 433091), Hs-TREM2-C1 (ACDbio, 420491) as part of the hybridization step. After the amplification step, sections were incubated with the TSA Plus fluorophores (TSA Cyanine Plus Evaluation Kit, Perkin Elmer, NEL744E001KT) for 30min at 40°C. Immediately after all steps were performed, sections were blocked and standard IHC was performed. Images were acquired with the Nikon A1R Eclipse confocal system.

### Aβ extraction and ELISA detection

Snap frozen brain tissue, collected as described above, was homogenizes with PBS in the presence of protease inhibitor using the FastPrep beads. Homogenates were centrifuged at 5000 g for 5 min and supernatants were collected and further centrifuged for 1 h at 100,000 g. Supernatant were recovered as PBS-soluble fraction. The insoluble material was then solubilized in 6 M guanidine buffer, sonicated and centrifuged at 70,000 rpm for 20 min, and labeled as guanidine-soluble fraction. The levels of Aβ40 and Aβ42 were measured by ELISA. First, ELISA Meso Scale Discovery (MSD) 96-well plates were coated with human antibodies for Aβ40 and Aβ42 at 4 °C overnight and blocked with 0.1% casein in PBS for 1 h. Next, standards and samples were loaded on to the plates together with the sulfo-tag labeled JRF/AβN/25 antibody. Plates were recorded on the MSD microplate reader.

### Statistical analyses

Statistical analyses were performed with Graphpad Prism 7 or 8 (LaJolla, Ca, USA). We compared experimental groups using Mann–Whitney test, one-way or two-way ANOVA (repeated measures) using Tukey’s multiple comparisons test as post hoc test. The differences from the MEA recordings were determined using one-way ANOVA with Dunnett's correction. Dose–response experiments were analyzed using a non-linear regression with a 95% confidence interval to calculate IC_50_ values with log-transformed ASO concentrations. The statistically significance levels were set at *p*-value < 0.05 (*), < 0.01 (**), < 0.001 (***), < 0.0001 (****) with a confidence interval of 95%. Data sets were plotted as mean ± standard deviation unless stated otherwise.

## Results

### Unbiased identification of ASOs targeting *APOE* and TREM2

To identify ASOs that target *APOE* and *TREM2* and reduce their levels via an RNase-H mediated knockdown, we designed 20-mer oligonucleotides tiling their respective pre-mRNA using a gliding window on their reference sequences without any restrictions. We utilized a 5–10-5 ASO configuration in which the 5 outer nucleosides contain 2’-O-methoxyethyl (2’MOE) modified riboses, whereas the central 10 nucleosides are deoxynucleosides. This design strategy resulted in 278 and 234 ASOs targeting the *APOE* and *TREM2* pre-mRNA, respectively. To test our candidate ASOs, we screened several immortalized cell lines for *APOE* and *TREM2* expression and determined that THP-1 cells highly expressed both target genes (Suppl Fig. 1C). Next, we treated THP-1 cells with a panel of *APOE* and *TREM2* ASOs for 72 h at a single concentration of 5 µM to identify candidate lead ASOs (Suppl Fig. 3A). In parallel, we used two different positive controls targeting either *MALAT1* RNA or total survival motor neuron (*SMN*) mRNA [70] as well as two non-targeting ASOs as negative controls [70] (Suppl Fig. 3B-E). We assessed *APOE* and *TREM2* mRNA levels using the 4 RT-qPCR assays where we excluded the assay(s) of which the ASO under investigation overlapped with the assay amplicon, as this may cause PCR interference. We identified 8 ASOs (out of 278; 2.8% hit rate) targeting *APOE* and 10 ASOs (out of 234; 4.2% hit rate) targeting *TREM2* resulting in at least 40% reduction of the target RNA at the single concentration tested (Suppl Fig. 3F-I). Next, we treated THP-1 cells with multiple concentrations (up to 20 µM) of the identified ASOs and confirmed that 4 *APOE* (ASO-1, ASO-13, ASO-86 and ASO-163) and 4 *TREM2* (ASO-33, ASO-171, ASO-192 and ASO-207) ASOs dose-dependently reduced *APOE* and *TREM2* APOE mRNA levels (Suppl Fig. 3J,K).

Ovareall, we identified 4 *APOE* and 4 *TREM2* ASOs that lead to dose-dependent reduction in target gene mRNA levels in THP-1 cell line.

### Pharmacological activity of *APOE* and *TREM2* ASOs in cultured human microglia

To confirm the efficacy of ASO treatment in human cells relevant to disease pathology, we used microglia-like cells (iMGL) [54, 55] derived from human induced pluripotent stem cells (iPSCs) [54, 55] (Suppl Fig. 4A,B) including homozygous APOE and TREM2 knockout (KO; APOE^−/−^ and TREM2^−/−^), homozygous APOE4 (APOE^4/4^) and homozygous TREM2 p.R47H (TREM2^R47H/R47H^) and isogenic control lines APOE3/3 and TREM2^WT^, respectively. We confirmed the correct differentiation of the lines (Suppl Fig. 4C-D, example APOE3/3 iMGL), and the expression of *APOE* and *TREM2* at the mRNA and protein levels in the various iMGL lines (Suppl Fig. 4E-I). We first explored if iMGLs were able to take-up ASOs by free delivery. We treated iMGLs with a 3’ or 5’ end fluorescently labelled or unlabeled *MALAT1* ASO (Suppl Fig. 5A). We observed a very rapid uptake of ASO reaching a plateau within 60 min after the addition of the ASO (Suppl Fig. 6A-F). We observed that the ASOs distributed to both the cytoplasm and nuclei after free delivery and that cytoplasmic ASOs accumulated into vesicular LAMP1-positive vesicles (Suppl Fig. 5B). We also confirmed that the uptake dynamics and ASO distribution were similar between different iMGL genetic backgrounds (Suppl Fig. 5B & 6A-F). Additionally, we assessed the pharmacological activity of ASOs in iMGL (APOE^ε3/ε3^ and APOE^ε4/ε4^ with a *MALAT1* ASO (Suppl Fig. 5C). *MALAT1* RNA levels were decreased with > 90% observed already at the lowest dose tested (Suppl Fig. 5D,E), and the levels were sustained until 10 days after ASO treatment (Suppl Fig. 5D,E).

**Fig. 1:**
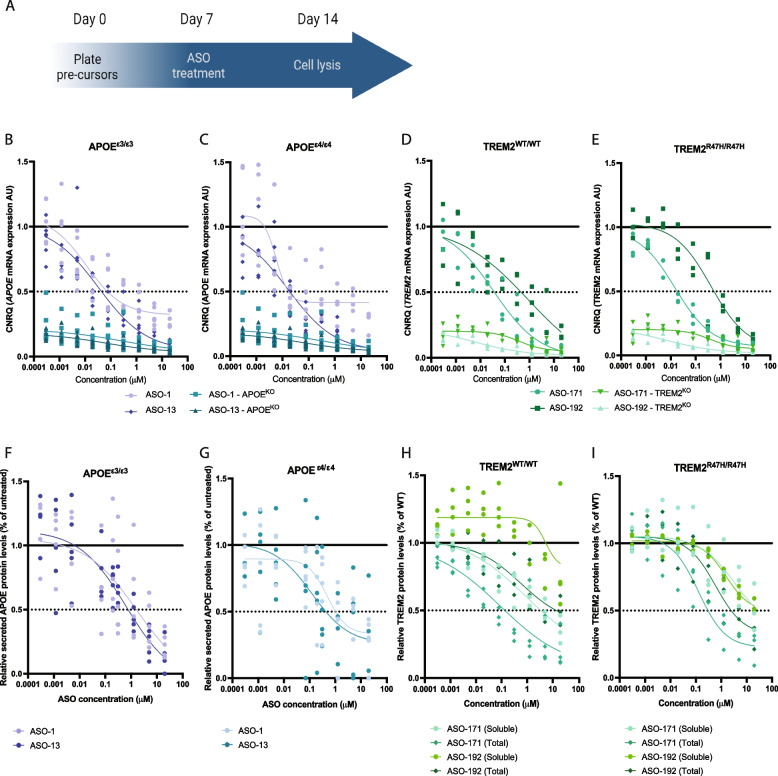
Target engagement of the lead APOE and TREM2 ASOs in cultured human microglia**. A** Experimental timelines for target engagement assessment of lead APOE and TREM2 ASOs in cultured iMGL. **B,C** Relative APOE mRNA levels were measured in cultured isogenic APOE^ε3/ε3^ (**B**) and APOE^ε4/ε4^ (**C**) iMGLs. Cultured isogenic APOE^KO/KO^ iMGL were used as control. All iMGLs were treated with APOE ASO-1 or ASO-13. CNRQ = Calibrated Normalized Relative Quantities. N = 5. **D,E** Relative TREM2 mRNA levels were measured in cultured isogenic TREM2^WT/WT^ (**D**) and TREM2^R47H/R47H^ (**E**) iMGLs. Cultured isogenic TREM2^KO/KO^ iMGL were used as control. All iMGLs were treated with TREM2 ASO-171 or ASO-192. CNRQ = Calibrated Normalized Relative Quantities. *n* = 3. **F,G** Relative secreted APOE protein levels medium were measured in culture media from isogenic APOE^ε3/ε3^ (**F**) and APOE^ε4/ε4^ (**G**) iMGLs treated with APOE ASO-1 or ASO-13. n = 6. **H,I** Relative soluble and total cellular TREM2 protein levels were measured in culture media and cellular extracts from cultured isogenic TREM2^WT/WT^ (**H**) and TREM2^R47H/R47H^ (**I**) iMGLs, respectively, treated with TREM2 ASO-171 or ASO-192. *n* = 4

We selected the two most potent *APOE* ASOs from the screen in THP-1 cells to evaluate their target engagement in cultured human microglia**.** We treated APOE^ε3/ε3^ iMGL with various doses of the two *APOE* ASOs (*APOE* ASO-1 and *APOE* ASO-13) 1 week after the initiation of microglial differentiation and evaluated *APOE* mRNA levels after 7 days (Fig. 1A). We observed a dose-dependent reduction following *APOE* ASO-1 and ASO-13 treatment with an IC_50_ of 11 and 33 nM, respectively (Fig. 1B**, **Suppl Table 6). We confirmed that the lead *APOE* ASOs were also effective in an APOE^ε4/ε4^ iMGL disease-relevant genetic background, with a very similar dose-dependent reduction of *APOE* mRNA (IC_50_ of 5.8 and 16 nM, respectively) (Fig. 1C**, **Suppl Table 6). The reduction of *APOE* mRNA was nearly complete at the RNA level based on a comparison with APOE^−/−^ iMGL (Fig. 1B,C). Next, we measured APOE protein levels using an ELISA-based method. APOE protein was not detectable or very close to detection limits in the cell lysates but was abundantly present in the culture medium at all time points suggesting very rapid secretion (Suppl Fig. 7A-D). We observed a dose-dependent reduction in APOE protein levels (IC_50_ of 1.5 and 0.6 µM in APOE^ε3/ε3^ iMGL and of 0.5 µM and 123 nM in APOE^ε4/ε4^ iMGL for ASO-1 and ASO-13, respectively) (Fig. 1F,G**, **Suppl Table 6). APOE protein levels were below detection limit in the APOE^−/−^ iMGL (Suppl Fig. 4F).

Next, we analyzed target engagement of the 2 most potent *TREM2* ASOs (*TREM2*-171 and *TREM2*-192) in iMGL. Both *TREM2* ASOs showed a similar dose-dependent pharmacological effect (IC_50_ = 35 and 880 nM, respectively) on *TREM2* mRNA levels (Fig. 1D**, **Suppl Table 6) and in the disease relevant TREM2^R47H/R47H^ iMGL (IC_50_ = 15 and 415 nM, respectively) (Fig. 1E**, **Suppl Table 6), with a reduction to TREM2^−/−^ levels at the highest concentration (Fig. 1D and E). In addition, we analyzed soluble (cleaved) and total (cellular) TREM2 from culture medium and cell lysates, respectively, 7 days after the addition of the ASOs using an ELISA-based detection method. ASO-171 showed a potent dose-dependent reduction of soluble and total TREM2 protein levels (IC_50_ = 842 nM and 89 nM, respectively), while ASO-192 was less effective (IC_50_ = 5.3 *µ*M and 581 nM, respectively) (Fig. 1H**, **Suppl Table 6). In disease relevant TREM2^R47H/R47H^ iMGL, ASO-171 showed a dose-dependent reduction of soluble and total TREM2 protein levels with IC_50_ = 3.6 µM and 129 nM, respectively, and ASO-192 with IC_50_ = 1.5 µM and 602 nM, respectively (Fig. 1I**, **Suppl Table 6). TREM2 protein levels were below detection limit in the TREM2^−/−^ iMGL (Suppl Fig. 4H, I).

These data demonstrate that our lead *APOE* and *TREM2* ASOs dose-dependently reduce the expression of their intended targets at RNA and protein level in cultured human microglia. As we observed higher potency of ASO-1, targeting APOE, and ASO-171, targeting TREM2, compared to ASO-13 and ASO-192, respectively, we used these ASOs in all subsequent experiments.

### ASOs are pharmacologically active in xenografted human microglia in the mouse brain

To prepare *APOE* and *TREM2* ASO efficacy studies in disease-relevant mouse models of Alzheimer’s disease, we explored if ASOs can also target disease-relevant genes in human microglia in vivo. To this end, we utilized a recently developed human microglia xenotransplantation model [48, 53]. We performed all in vivo experiments using the H9 human embryonic stem cell line (hESC) coupled to the MIGRATE differentiation protocol, which was optimized for an efficient and consistent transplantation [48, 53]. We first confirmed that our lead ASOs targeting *APOE* and *TREM2*, as well as the MALAT1 positive control, are also effective in reducing expression of their target genes in a dose-dependent manner in H9-hESC derived microglia after differentiation using MIGRATE (Suppl Fig. 8).

**Fig. 2:**
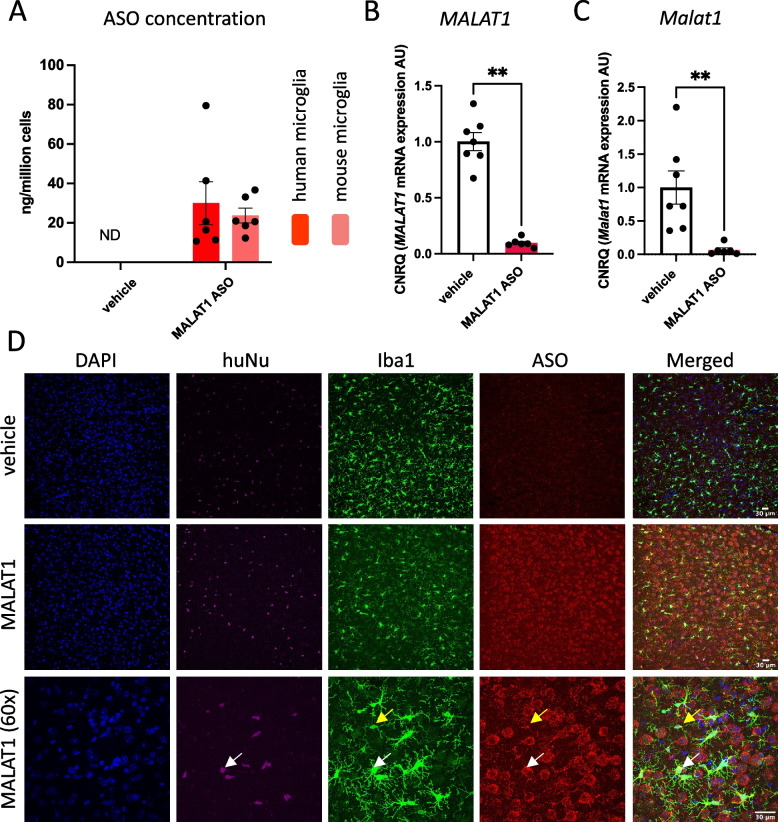
ASOs are internalized and pharmacologically active in human microglia xenografted in mouse brain.** A** ASO concentration in human and mouse microglia isolated from 10–12-week-old xenotrasplanted mice 7 days after administration of 125µg MALAT1 targeting ASO. **B,C** Expression of human MALAT1 (**B**) and mouse Malat1 (**C**) in isolated human and mouse microglia, respectively, from mice treated with MALAT1 ASO compared to vehicle treated mice. **D** Representative images of widespread ASO distribution 7 days post MALAT1 ASO treatment. Human microglia were identified as Iba1 and huNu positive cells, and mouse microglia as Iba1 positive and huNu negative cells. Nuclei were visualized with DAPI. White arrow indicates an example of human microglia, while yellow arrow indicates a mouse microglia. Scale bars represent 30µm. CNRQ = Calibrated Normalized Relative Quantities. Bars represent mean ± SEM, n = 5–7/group. Statistical differences based on the Mann–Whitney test: **p < 0.01

Next, we determined if ASOs can also be taken up by and exert their function in human microglia in the brain. Ten to twelve weeks after transplantation, we delivered a single bolus dose of the positive control ASO targeting *MALAT1* or vehicle via bilateral intracerebroventricular (icv) injection. One week after the injection, we used 3 complementary approaches to assess if the ASO was present and pharmacologically active in the xenografted human microglia. We analyzed *MALAT1* ASO levels in human and mouse microglia isolated by flow cytometry (CD11b^+^ hCD45^+^ and CD11b^+^ mCD45^+^ cells, respectively, Suppl Fig. 2) from the same brains. We measured ~ 30 ng/1 × 10^6^ cells and ~ 24 ng/1 × 10^6^ cells of the *MALAT1* ASO in human and mouse microglia, respectively (Fig. 2A). We also observed a reduction of 90% in *MALAT1* levels in human microglia compared to microglia isolated from vehicle-treated animals (Fig. 2B**)**. Similarly, the mouse *Malat1* transcript was reduced up to around 95% in isolated mouse microglia (Fig. 2C). These data indicate that ASOs are taken up and pharmacologically active in endogenous mouse microglia as well as xenografted human microglia in vivo. Finally, we explored if the *MALAT1* ASO was taken up by human microglia throughout the xenografted mouse brain. Using an antibody raised against the ASO backbone, we confirmed the ASO distribution by IHC across multiple regions of the brain including cortex, striatum, thalamus as well as CA1, CA3 and dentate gyrus regions of the hippocampus (Suppl Fig. 9A). Co-localization of the ASO with a human-specific nuclear marker confirmed that the ASO was internalized by human microglia, but also by endogenous microglia and other brain cells (Fig. 2D).

Taken together, these data demonstrate that ASOs are pharmacologically active in human microglia in vivo when delivered directly into the CNS.

### Dose-dependent APOE and TREM2 ASO-mediated reduction of target RNA expression in human microglia in the brain.

**Fig. 3:**
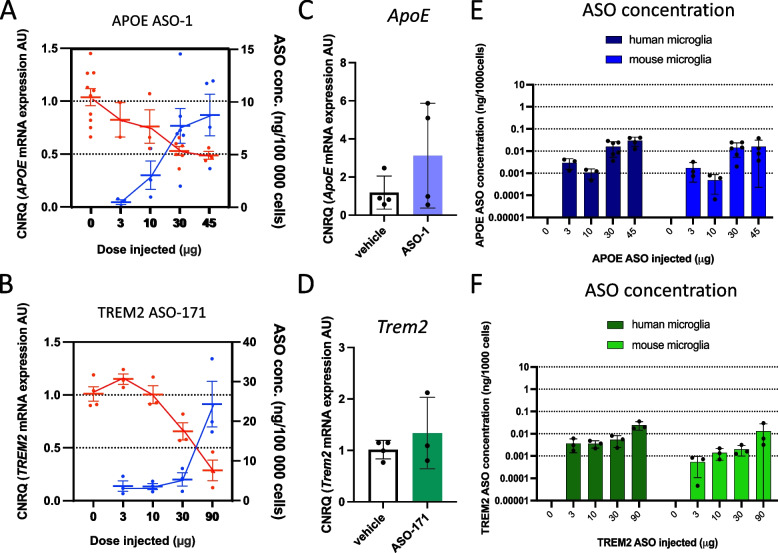
(next page). Dose-dependent activity of APOE and TREM2 ASOs in human microglia xenografted in mouse brain.** A,B** Dose-dependent reduction in APOE (**A**) and TREM2 (**B**) expression in human microglia isolated from 10–12-week-old xenotransplanted mice 7 days after administration of APOE ASO-1 (**A**) or TREM2 ASO-171 (**B**) (red dots/line-knockdown level, blue dots/line-ASO concentration). (**C,D**) Expression of mouse ApoE (**C**) and Trem2 (**D**) in isolated mouse microglia after treatment with 45µg APOE ASO-1 (**C**) or 90µg TREM2 ASO-171 (**D**). CNRQ = Calibrated Normalized Relative Quantities Dots or bars represent mean ± SEM, *n* = 2–10/group. **E–F** ASO concentrations in human and mouse microglia isolated from 10–12-week-old xenotransplanted mice 7 days after administration of APOE ASO-1 (**E**) or TREM2 ASO-171 (**F).** Mean + SD, *n* = 3

We assessed if our *APOE* and *TREM2* ASOs could induce an RNase-H mediated knockdown of *APOE* and *TREM2* in human microglia in vivo in the *App*^*NL−G−F*^ model of AD. We first confirmed that *APOE* and *TREM2* are expressed in xenografted human microglia at the RNA level and increased with amyloid-β exposure, and mainly localized in the DAM population (Suppl Fig. 10). We treated xenografted mice with a bilateral icv injection with various doses of the *APOE* ASO-1 and *TREM2* ASO-171 and compared their activity to PBS injected controls. Since we observed mice did not tolerate high doses of *APOE* ASO-1, we lowered its dose (ranging from 3 to 45 µg) compared to the *TREM2* ASO-171 (ranging from 3–90 µg). One week after the injection, we isolated human and mouse microglia from the xenografted brains by flow cytometry (CD11b^+^ hCD45^+^ and CD11b^+^ mCD45^+^ cells, respectively, Suppl Fig. 2) and measured ASO, as well as *APOE* and *TREM2* mRNA levels. Treatment with the human-specific *APOE* ASO-1 resulted in a dose-dependent knockdown of *APOE* mRNA levels in isolated human microglia with a maximal reduction of ~ 50% at the highest dose tested (45 µg) (Fig. 3A). The degree of knockdown was consistent with the ASO levels measured in the isolated cells (Fig. 3A). Although we observed similar ASO levels in isolated mouse microglia, we could not detect any significant changes in mouse *ApoE* mRNA levels, confirming the specificity of ASO-1 for the human target transcript (Fig. 3C and E). *TREM2* ASO-171 treatment also resulted in a dose-dependent reduction of *TREM2* mRNA levels up to ~ 70% at the highest dose tested (90 µg) compared to *TREM2* levels in human microglia isolated from vehicle treated mice (Fig. 3B**).** *TREM2* ASO-171 levels in isolated human microglia were also consistent with its pharmacological activity (Fig. 3B). Similar ASO levels were measured in isolated mouse microglia, but mouse *Trem2* mRNA levels remained unaffected (Fig. 3D and F**)**. Correlating the measured exposure levels of both ASOs with their pharmacological effect indicated that approximately 10 ng/1 × 10^5^ cells of ASO is required to reduce their target RNA by 50% (Fig. 3A,B).

In summary, we demonstrated that ASOs targeting disease-relevant microglial genes are pharmacologically active in human microglia after direct CNS delivery.

### ASO-mediated APOE and TREM2 knockdown leads to a shift in cell states in response to amyloid pathology.

**Fig. 4:**
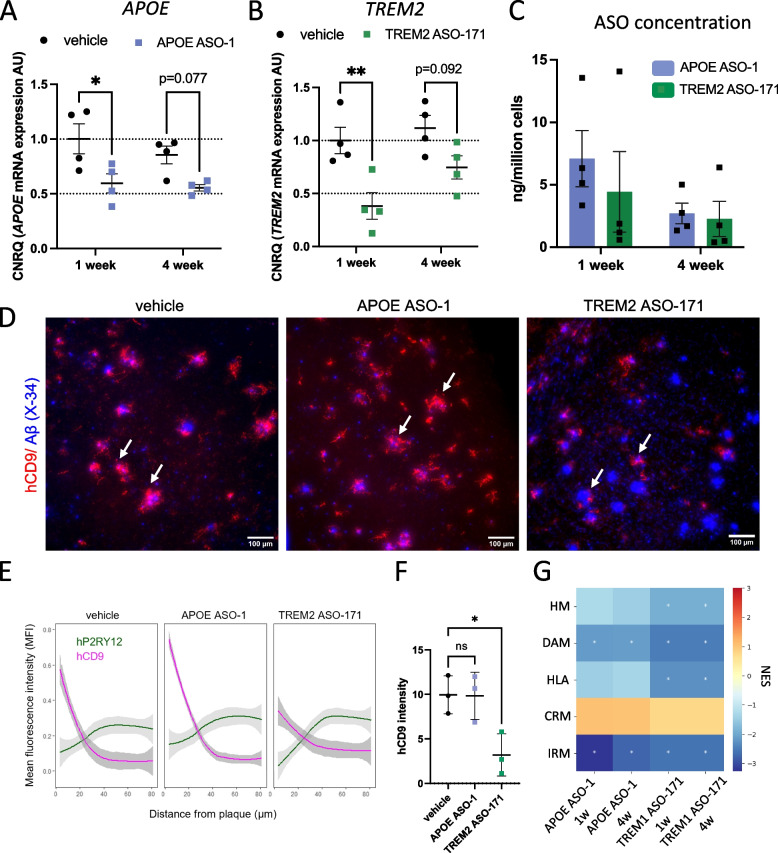
Phenotypic and transcriptomic changes in human microglia upon ASO mediated APOE and TREM2 knockdown.** A,B** APOE (**A**) and TREM2 (**B**) expression in human microglia isolated from 6-month-old App^NL−G−F^ mice treated with 45ug APOE ASO-1 (**A**) or 90ug TREM2 ASO-171 (**B**) for 1 or 4 weeks. **C** ASO concentration in human microglia isolated from 6-month-old App^NL−G−F^ mice treated with APOE ASO-1 or TREM2 ASO-171 for 1 or 4 weeks. CNRQ = Calibrated Normalized Relative Quantities. Dots or bars represent mean ± SEM, n = 4/group. Statistical differences based on Two-way ANOVA test: *p < 0.05, **p < 0.01. **D** Representative images showing activated human microglia targeting X-34 positive amyloid-β fibrils 4 weeks post APOE and TREM2 knockdown. Arrow indicates an instance of activated human microglia around plaques. Scale bars = 100 µm (**E**) Quantification of mean fluorescence intensity for hP2RY12 and hCD9 at varying distances from the plaque assessed 4 weeks following ASOs treatment. **F** Quantification of hCD9 intensity at the plaque site (10 µm) normalized against signal intensity at the distal site from the plaque (74 – 81 µm) 4 weeks post ASOs treatment. Analysis was restricted to plaques surrounded by engrafted human microglia, determined by hP2RY12 signal (see Suppl Fig. 11A). Data represented as mean ± SD, n = 3/group. Statistical significance was evaluated with One-way ANOVA test (*p < 0.05, ns, not significant). **G** Gene set enrichment analysis (GSEA): Pre-ranked results of the microglial subtype gene sets (top 50 most significantly up-regulated genes of each microglial subtype [22]). The color represents the Normalized Enrichment Score (NES) of each gene set against the full list of genes ranked by the log-fold change when comparing the treatment group against the vehicle group. The asterisk indicates gene sets that are significantly enriched with FDR *p < 0.05. All analysis were performed on xenotransplanted microglia isolated from 6–7-month-old App^NL−G−F^ mice treated with APOE ASO-1 or TREM2 ASO-171 for 1 or 4 weeks

We recently characterized in-depth the response of xenografted human microglia to amyloid-β pathology in *App*^*NL−G−F*^ mice. We reported that they acquire a wide range of transcriptional states in response to different forms of soluble and insoluble amyloid-β, some of them not previously observed in mouse systems [15, 16, 48, 62, 71]. Therefore, we assessed whether the knockdown of *APOE* and *TREM2* by ASOs results in a shift in transcriptional states in transplanted human microglia in response to amyloid-β pathology in the mouse brain. We first determined if ASO treatment results in sustained knockdown of microglial target genes in human microglia for longer periods. We treated 6 months old xenografted mice with either 45 µg of *APOE* ASO-1 or 90 µg of *TREM2* ASO-171 and isolated human microglia either 1 week or 4 weeks after the bolus icv injection. Treatment with the *APOE* ASO-1 resulted in sustained lowering of *APOE* expression levels over a period of 4 weeks (~ 40% and ~ 35% at 1 and 4 weeks after treatment, respectively, compared to vehicle treated group) (Fig. 4A). *TREM2* ASO-171 treatment resulted in a strong reduction of *TREM2* mRNA levels after 1 week (~ 60% reduction compared to vehicle treated group) but its expression levels were partially recovered after 4 weeks (~ 30% reduction compared to vehicle treated group) (Fig. 4B). We did not observe significant differences in the level of ASO between any group or time point (Fig. 4C). To evaluate the response of human microglia to amyloid-β plaques upon knockdown of *APOE* and *TREM2*, we assessed microglia accumulation around amyloid-β plaques and the phenotypic transition to DAM by measuring the intensity of hCD9, marker of DAM signature, around plaques in xenotransplanted AD mouse brains at 1 and 4 weeks after ASOs injection. While no differences were found at 1-week post-treatment (Suppl Fig. 11B-D), we observed reduced response of human microglia to pathology upon *TREM2* knockdown at 4 weeks after ASO-171 injection compared to vehicle treated mice evident by reduced hCD9 signal (Fig. 4D-F). In contrast to ASO-171, we did not observe any changes in hCD9 signal around amyloid-β fibrillar inclusions 4 weeks after *APOE* ASO-1 treatment (Fig. 4D-F).

To determine whether this effect on phenotype was also correlated with a transcriptomic shift of the microglia, we performed bulk RNA sequencing on isolated microglia and found > 2000 and > 4500 differentially regulated genes in *APOE* ASO-1 and *TREM2* ASO-171 treated mice, respectively, compared to PBS injected controls (Suppl Tables 7 ,8). Out of these differentially regulated genes, approximately 50% were upregulated and 50% were downregulated. We investigated whether the treatment with ASOs specifically altered the expression of genes from different microglial responsive states, including homeostatic (HM), disease associated (DAM), antigen presenting (HLA), cytokine response (CRM) and interferon response microglia (IRM) [22]. We performed gene enrichment analysis taking the top 50 markers of each microglia cell state and confirmed that human microglia treated with *TREM2* ASO-171 showed a downregulation of DAM and HLA genes (Fig. 4G). Interestingly, we also observed that the *APOE* ASO-resulted in a downregulation of DAM microglia signature, which was not reflected in the histological analysis (Fig. 4D-G). Looking more closely into the top 10 marker genes of each microglia transcriptional phenotype, we confirmed that the *TREM2* ASO-171 mainly affected DAM and HLA cluster genes, whereas the *APOE* ASO-1 affected only DAM cluster genes (Suppl Fig. 12E). Furthermore, we observed that downregulation of DAM and HLA genes 1 week after *TREM2* ASO-171 treatment slowly diminished after 4 weeks, corresponding to the lower ASO knockdown efficiency 4 weeks after treatment (Fig. 4B and Suppl Fig. 12E). While we also observed an upregulation in a subset of CRM cluster genes with both *APOE* ASO-1 and *TREM2* ASO-171 (Supplementary Fig. 12E), the changes were non-significant in the gene enrichment analysis (Fig. 4G).

In summary, we demonstrated that ASOs targeting *APOE* and *TREM2* lead to rapid transcriptional change in the transplanted human microglia, that was followed by reduction in the response of these cells to amyloid-β plaques after 4 weeks of treatment.

### Human microglia with ASO-mediated APOE and TREM2 knockdown resemble APOE-KO and TREM2-KO microglia

We explored whether transcriptional changes in microglia induced by ASO-mediated *TREM2* and *APOE* and knockdown in microglia are consistent with previously published data on TREM2- KO and APOE-KO microglia. Studies in mice and previous data from our lab in xenotransplantation models have shown that both the deficiency of *TREM2* and *APOE* can alter the transcriptional response of microglia to amyloid pathology [15, 16]. We recently showed that *TREM2*-KO xenografted human microglia lose the ability to transition into DAM and HLA phenotypes and stay vastly in a homeostatic state [22]. To explore if transcriptional changes in human microglia caused by the ASO treatment mimic those in *TREM2*- and *APOE*-KO microglia, we compared the effects we observed after ASO administration (vs. PBS vehicle injected controls) with the transcriptional alterations in TREM2-KO and APOE-KO (vs. isogenic wild-type controls) human microglia transplanted in *App*^*NL−G−F*^ mice [22].

**Fig. 5:**
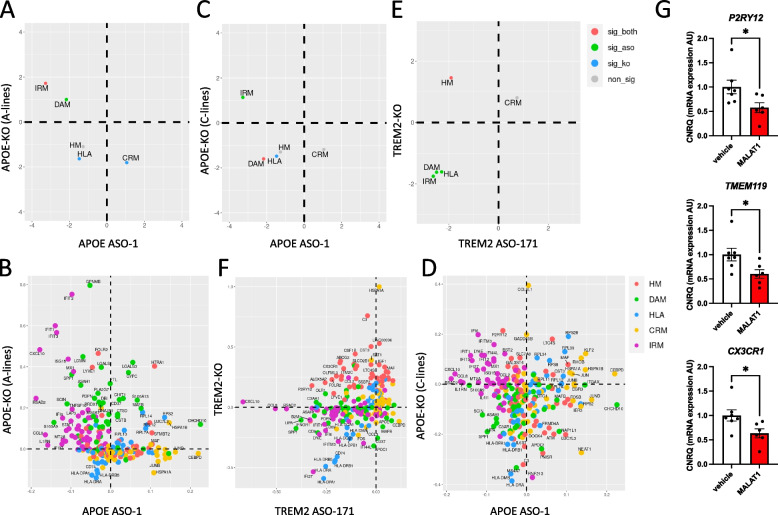
Comparison of transcriptional profiles between ASO mediated APOE and TREM2 knockdown with full APOE-KO and TREM2-KO human microglia.** A,C,E** Gene set enrichment analysis (GSEA): Pre-ranked enrichment scores of the microglial subtype marker gene sets under knockout and ASO treatment. Each point represents the GSEA Normalised Enrichment Score of the gene set (top 50 marker genes of the labelled microglial subtype) against the full list of genes ranked by the log-fold change when comparing the ASO treatment group against the vehicle group (x-axis) and the knockout group against the control group (y-axis). Significant GSEA enrichment is denoted by FWER < 0.05. **B** Scaled log fold changes in gene expression between APOE knockout and control A-lines (y-axis), and APOE ASO-1 treatment and the vehicle at 1 week (x-axis) of the top 50 marker genes from each microglial subtype. **D** Scaled log fold changes in gene expression between APOE knockout and control C-lines (y-axis), and APOE ASO-1 treatment and the vehicle at 1 week (x-axis) of the top 50 marker genes from each microglial subtype (**F**) Scaled log fold changes in gene expression between TREM2 knockout and control (y-axis), and TREM2 ASO-171 treatment and the vehicle at 1 week (x-axis) of the top 50 marker genes from each microglial subtype. Since the Knockout experiment and the ASO treatment experiments were performed under different experimental conditions, the Log2fold change of each DE analysis was scaled to -1 and 0 for LFC < 0 and 0 to 1 for LFC > 0. All analysis were performed on xenotransplanted microglia isolated from 6–7-month-old App^NL−G−F^ mice. **G** Expression of microglia homeostatic markers, P2RY12, TMEM119 and CX3CR1 in human microglia isolated from 10–12-week-old xenotrasplanted mice 7 days after administration of 125ug MALAT1 targeting ASO compared to vehicle treated mice. CNRQ = Calibrated Normalized Relative Quantities. Bars represent mean ± SEM, *n* = 5–7/group. Statistical differences based on the Mann–Whitney test: *p < 0.05

We observed that both genetic ablation and ASO-mediated knockdown of *TREM2* resulted in a downregulation of DAM and HLA genes, but also IRM genes (Fig. 5E,F). However, treatment with TREM2 ASO-171 only partially phenocopies a full loss-of-function, as it failed to mimic the shift towards a homeostatic state we previously observed in TREM2-KO cells (Fig. 5E,F). We were not able to recapitulate an increase in HM observed in TREM2-KO microglia and we report a downregulation by *TREM2* ASO-171 treatment (Fig. 5E,F). We speculate downregulation in HM might arise from non-specific ASO effects, as we have a similar effect after the administration of control ASO MALAT1 at 125ug (Fig. 5G).

On the other hand, ASO-mediated knockdown of *APOE* resulted in a slight downregulation of HLA genes, which was significant in *APOE*-KO microglia, but not after ASO-mediated knockdown (Fig. 5A, B, C and D). In addition, treatment with *APOE* ASO-1 resulted in a partial downregulation of DAM genes, which was consistent with our previous observations in APOE-KO C-lines (Fig. 5C and D), but not in the APOE-KO A-lines (Fig. 5A and B). CRM and IRM followed an opposite trend in the *APOE* ASO-1 treated microglia, in comparisons to both APOE-KO A- and C-lines (Fig. 5A, B, C and D).

Overall, we demonstrate that in vivo ASOs targeting *APOE* and *TREM2* lead to transcriptional changes in human microglia that are partially comparable to those observed in APOE- and TREM2-KO human microglia.

### Treatment with ASOs targeting APOE and TREM2 does not affect amyloid burden in App^NL−G−F^ mice

**Fig. 6:**
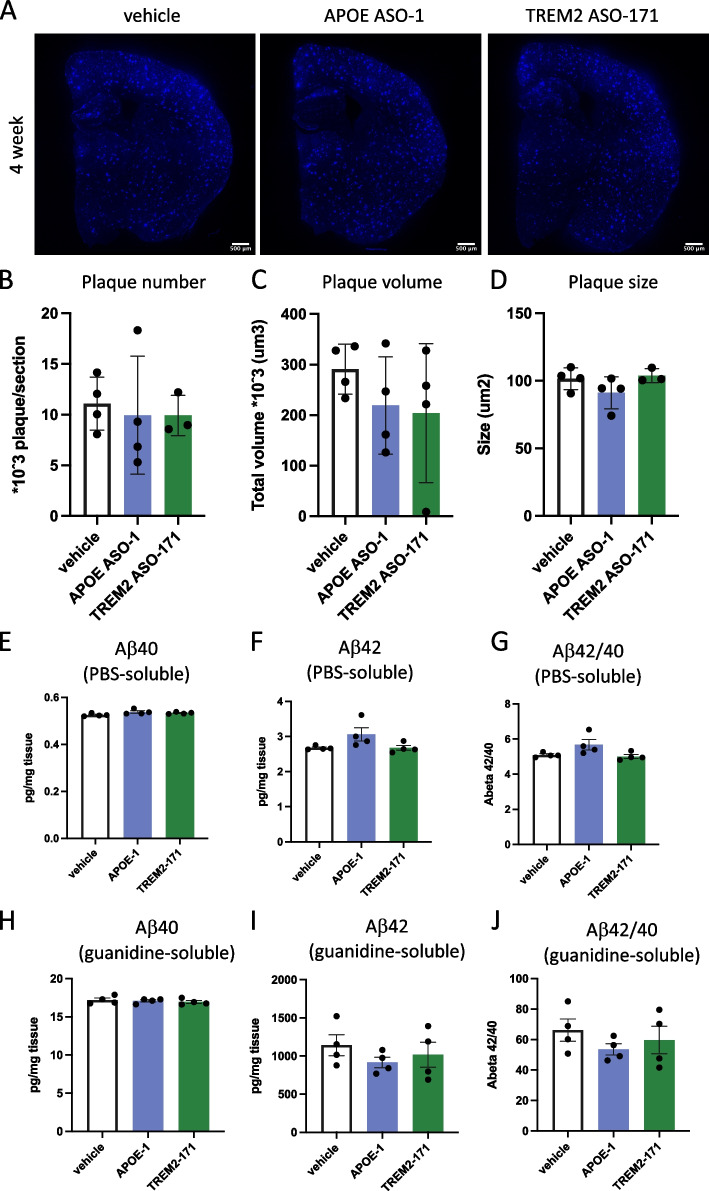
Treatment with ASO’s targeting APOE and TREM2 does not affect amyloid burden in APP^NL−G−F^ mice.** A** Representative images of immunofluorescent analysis of amyloid plaques. Plaques were visualized with X-34. Scale bar represents 500um. **B-C** Quantification of X34-stained amyloid plaques; plaque numbers (**B**), total plaque volume (**C**) and average plaque size **D** was analyzed. **E–G** Levels of Ab40 (**E**) and Ab42 (**F**), and Ab42/40 (**G**) ratio in PBS-soluble fraction of brain homogenates. **H-J** Levels of Ab40 (**H**) and Ab42 **(I),** and Ab42/40 **(J)** ratio in guanidine-soluble fraction of brain homogenates. Bars represent mean ± SEM, *n* = 4/group

To assess if the transcriptional and phenotypical microglia alterations induced by ASO mediated knockdown affected amyloid pathology, we assessed the levels of amyloid-β in *App*^*NL−G−F*^ mice treated for 4 weeks with APOE-1 and TREM2-171. We quantified the number of X34 + amyloid-β plaques and total plaque volume, as well as individual plaque size by immunohistochemistry but did not observe any impact of APOE-1 and TREM2-171 treatment (Fig. 6A, B, C and D). We also assessed the levels of Aβ40 and Aβ42 in brain homogenates after separating the PBS- and guanidine-soluble fractions. In line with the plaque quantification, we observed no differences in either PBS- or guanidine-soluble Aβ42 or Aβ40 after the APOE-1 or TREM2-171 treatment (Fig. 6E, F, G, H, I and J**)**. These data show that even though ASOs targeting *APOE* and *TREM2* lead to transcriptional and phenotypical changes in human microglia 4 weeks after treatment, they are not able to alter amyloid pathology at the time point investigated here.

### Safety profiles of the lead APOE and TREM2 ASOs

Finally, we performed a series of early safety assessments on our candidate lead *APOE* and *TREM2* ASOs to determine feasibility for their potential use as therapeutic tools. We focused on 3 known liabilities for ASOs in general and for their application for CNS delivery in particular [41, 65, 72–75].

First, we evaluated potential transcriptional off-target effects by treating cultured WT iMLG with 2 different concentrations (1.25 µM and 20 µM) of our candidate lead *APOE* (*APOE-1*) and *TREM2* (*TREM2-171*) ASOs for 24 and 48 h. Transcriptional changes were evaluated comparing the lead ASOs to the untreated condition with microarray (Suppl Fig. 13A-B, Suppl Table 8, 9, 10 and 11). Importantly, we looked at the differentially expressed genes (DEGs) at both time-points for each ASO at each dose and considered the DEGs present in both 24 and 48 h the true off-target genes. The expression of a larger number of genes was altered after 24 h of treatment, especially for the APOE-1 ASO. As these DEGs are not conserved at the later timepoint, they reflect acute responses to the ASO treatment itself rather than off-target effects caused by silencing APOE.

The *APOE* ASO-1 with a 1.25 µM dose had 9 DEGs and with a 20 µM dose 194 DEGs at both timepoints (Suppl Fig. 13A, Suppl Table 8). The *TREM2* ASO-171 ASO at a 1.25 µM dose had 1 DEG and with a 20 µM dose 11 DEGs at both timepoints (Suppl Fig. 13B, Suppl Table 9).

In addition, we looked at the conserved DEGs between the 2 ASOs targeting *APOE* (APOE-1 vs. APOE-13, Suppl Tables 8 and 10) or *TREM2* (TREM2-171 vs. TREM2-192, Suppl Tables 9 and 11). The DEGs that are conserved between the 2 ASOs are annotated in red in the tables and are considered to be the true off-target genes. For the APOE ASOs, upon a 20 µM dose and conserved over the timepoints, 13 out of 194 DEGs are conserved (Suppl Tables 9, 10 and 11). For the TREM2 ASOs, upon a 20 µM dose and conserved over the timepoints, 2 out of 11 DEGs are conserved (Suppl Table 9–11). Since the *APOE* ASO has a larger number of overlapping DEGs with the 20µM dose, we looked further into another potential liability for ASOs. It has been shown that specific ASO sequences can elicit seizures when administered centrally in animals [75], and therefore we assessed the seizurogenic potential after 90 min of incubation with various concentrations of the *APOE* ASO-1 and *TREM2* ASO-171 using a panel of predefined electrophysiological properties measured in rat primary neuron cultures using a multi-electrode array [60] (Suppl Fig. 13C-D). Treatment with *APOE* ASO-1 resulted in decreased action potential weighted mean firing rates, decreased number of neural network bursts and a decreased area under cross correlation and were significant at higher concentrations (starting at 1 µM) (Suppl Fig. 13C). Burst duration at all concentrations was not affected except at the highest concentration of 10 µM (Suppl Fig. 13C**)**. The observed changes were consistent with an inhibitory/sedative CNS hazard. Treatment with various concentrations of *TREM2* ASO-171 did not elicit any significant dose-dependent electrophysiological alterations that may represent a CNS hazard (Suppl Fig. 13D).

Finally, we determined the immunogenicity of our ASOs by measuring the levels of proinflammatory cytokines in wild-type and disease-relevant iMGL after a 24h treatment (Suppl Fig. 14A,B and Suppl Fig. 15A,B). We used lipopolysaccharide (LPS) as positive control. The *APOE* and *TREM2* ASOs did not induce the secretion of cytokines (Suppl Fig. 14A,B and Suppl Fig. 15A,B). We confirmed these findings in freshly isolated human whole blood from 4 independent donors, using TLR-7 and TLR-8 agonists as positive control [76] (Suppl Fig. 14C).

In summary, our lead *TREM2* ASO is highly selective for its intended target, while the *APOE* ASO has several off-target genes upon treatment with a higher dose in cultured human microglia. Our ex vivo studies did not indicate any liabilities that would preclude initial testing in vivo for the *TREM2* ASO, although the *APOE* ASO did show a sedative effect at higher doses. Finally, our lead ASOs do not elicit a proinflammatory response by human microglia.

## Discussion

Neuroinflammation contributes to the pathogenesis of several neurodegenerative diseases including AD. As resident innate immune cells of the CNS, microglia play key roles in the process of neuroinflammation and disease-associated variants in several microglial genes including *APOE* and *TREM2* confer increased risk to AD. The development of APOE and TREM2 therapeutics has been hindered by their expression both in the peripheral and brain-resident immune cells [77, 78]. Translational research is also hampered by the low species evolutionary conservation of microglial gene sequences and protein functions [49, 50]. In this study, we show that ASOs are taken up readily by human cultured microglia and that they are pharmacologically active at very low concentrations in microglia after unfacilitated free delivery. We identified potent *APOE* and *TREM2* ASOs that dose-dependently reduce the expression levels of their intended target in cultured human microglia. We show that ASOs are also effectively taken up by human microglia grafted in mouse brains. Delivery of *APOE* and *TREM2* ASOs into the CSF of adult mice resulted in a dose-dependent reduction of their intended target in xenografted human microglia with ASOs showing the potential to lower their target RNA expression for at least 4 weeks. Downregulation of *APOE* and *TREM2* with ASOs resulted in significant changes in the transcriptional response of microglia to amyloid-β pathology in vivo, which is partially consistent with the phenotypic alterations observed in APOE- or TREM2-KO cells. Finally, safety profiles of the lead ASOs show no safety hazards for the *TREM2* ASO, while the *APOE* ASO has several off-target genes and a sedative effect at the higher doses.

### Pharmacological activity of antisense oligonucleotides in human microglia

Prior studies have demonstrated that ASOs and siRNAs can reduce their target RNA expression levels in mouse microglia by formulating the oligonucleotides with complex lipid- or peptide-based conjugations or formulations such as lipid-based nanoparticles [79–81]. In humans, ASOs appear to co-localize with various cell types present in the CNS including microglia following a single or repeated intrathecal injections suggesting that ASOs can enter these cells in humans as well [38]. Whether ASOs are also pharmacologically active in human microglia was not known. Our data indicate that *APOE* and *TREM2* ASOs can enter human microglia and exert their function both in vitro and in vivo after delivery into the CSF in mouse CNS without the need of complex and expensive conjugation and formulation strategies.

Although both passive and active receptor-mediated uptake mechanisms have been identified in cellular models [82], the mechanisms by which ASO are taken up by microglia have not been specifically investigated. Future experiments using human iPSC-derived microglia will help to shed light on the mechanisms governing microglial ASO uptake. These experiments can potentially identify targeted ASO therapeutics approaches to modulate expression of microglial genes with enhanced cell type specificity and potency and less off-target effects in other non-targeted cell types or tissues.

Microglia can recognize potentially pathogenic RNA and DNA molecules with either membrane-bound or intracellular pattern recognition receptors (PRRs) [83–85]. As ASOs represent chemically modified hybrid RNA::DNA molecules, they could be recognized by microglial PRRs as potential substrates and they may cause an immunogenic reaction [86, 87]. Our early safety assessment demonstrated that cultured human microglia do not elicit a cytokine storm when they are acutely exposed to our lead *APOE* and *TREM2* ASOs. Thus, our ASOs targeting microglial genes are not immunogenic in nature. This is also consistent with the chemical modifications we used when designing our ASOs. The phosphorothioate backbone and the methylated state of the 5 position of cytosines throughout the entire oligonucleotide reduces the risk of immunogenicity as has been reported by others [88–90]. These chemical modifications are now considered the gold standard and are also validated to be safe and tolerated for direct CNS delivery in humans [38, 39, 91, 92].

### Human versus mouse microglia

Recent advances in RNA sequencing technology including single-cell and single-nucleus RNA sequencing have provided important insights into species differences at the genetic and transcriptomic level [49–51] and revealed that ~ 40% of the human genes lack a clear mouse ortholog [52]. A similar observation was made when evaluating AD-associated risk loci [48]. Importantly, several key human microglial genes genetically associated with AD showed low species conservation at the protein level [48]. Many groups have also described microglia in mouse models of AD that obtain a ‘disease-associated microglia’ (DAM) state during the development of their phenotype [16, 93]. Human microglia in human AD brains acquire a much more complex range of transcriptomic signatures that include, and go beyond the disease-associated states reported in mouse systems [94–96]. These data indicate that human and mouse microglia are genetically and functionally different and highlight the importance of evaluating therapeutic modalities including ASOs in a human genetic and cellular context. Despite these differences, we found that ASOs are internalized at similar levels in both human and mouse microglia in mouse brains suggesting that the microglial ASO uptake mechanisms are conserved between these species. We identified and characterized ASOs that target human specific *TREM2* and *APOE* as demonstrated by the robust target RNA knockdown and concomitant lack of expression changes of their mouse ortholog RNA in mouse microglia. Our ASOs can thus be used in future experiments to assess the functional consequences of reducing human *TREM2* and *APOE* expression in human microglia selectively.

### ASO treatment can modify human microglial cell states in vivo

It is well established that both APOE and TREM2 play major roles in microglia physiology and function, particularly in the context of neurodegeneration. Loss of function in APOE prevents microglia from acquiring a neurodegenerative phenotype [15, 16], while TREM2 knockout locks microglia in homeostatic state, preventing them from transitioning to DAM or HLA phenotypes [15, 16, 22]. However, it is still not completely clear how these transcriptional changes influence AD pathology. TREM2 modulation, for example, has been a topic of discussion in recent years, as studies so far have reported contradictory results. However, it is clear that effects resulting from TREM2 deletion are largely dependent on the stage of the disease [97–99]. Here, we profiled ASO-mediated knockdown approach, which gives us an opportunity to control the timing, but also the level of *APOE* and *TREM2* inhibition. By titrating the dose of ASO, we were able to see as much as 50–70% of target gene inhibition. We then applied this approach in 6-month-old *App*^*NL−G−F*^ mice xenotransplanted with human microglia, to determine if we could alter microglia transcriptome after acute manipulation of *APOE* and *TREM2* when amyloid-β pathology is already present in the brain [22].

We observed that acute downregulation of *APOE* and *TREM2* by ASOs was sufficient to induce significant transcriptional changes in microglia. Reduction of both *APOE* and *TREM2* with ASOs lowered the expression of HLA genes, which indicates that ASOs targeting AD risk genes can diminish microglia activation even in the presence of amyloid-β pathology. Interestingly, the changes we observed after ASO treatment were not completely consistent to those reported in xenotransplanted *APOE-* and *TREM2*-KO microglia [22]. We reason that any differences between the two approaches could arise from 1) different levels of inhibition (i.e. partial inhibition versus full inhibition), 2) time of inhibition, (i.e. chronic, from birth versus acute, at 6 months of age, or 3) non-specific effects elicited by ASO treatment or induced by the constitutive absence of either APOE or TREM2. Nevertheless, ASOs provide a valuable tool to study the effects of partial gene knockdown as well as timely gene inhibition. These characteristics open avenues to more targeted therapeutic strategies to modulate microglial biology and provide new tools to answer the question as to what the contribution of neuroinflammation in AD pathology is.

### Is reducing APOE and *TREM2 *expression in central myeloid cells therapeutically relevant?

Our data demonstrate that *APOE* and *TREM2* ASOs can be used to study the role of these microglial AD risk genes in human microglia in vivo. First, icv bolus delivery of *APOE* and *TREM2* ASO in CSF of xenografted mice showed an extensive distribution of the ASOs in the CNS. Our data are consistent with the uniform distribution of ASOs throughout the CNS after delivery in the CSF in both rodent and non-rodent preclinical models [34–36]. Second, while the ASOs were designed to target their human target RNA, they are taken up by many cell types in the CNS including in the human xenografted microglia as well as the endogenous mouse microglia. Our data are in line with a rodent study that measured the effective ASO doses in various cell types of the CNS [36]. This study demonstrated that microglia show effective doses that are 2–3 times lower than the effective dose required for neurons [36]. Finally, the pharmacological effect of the ASOs is lowered, but still detectable in xenografted human microglia 4 weeks after the bolus CSF delivery. The long-lasting effect of ASOs after a single direct CNS delivery has also been observed in humans after an intrathecal procedure [38, 39, 91, 92]. However, recent study reports striking differences in duration of ASO action between different cell types in the brain, with microglia having shortest ASO activity [100]. In line with this study, we do observe a substantial wash-out of TREM2-targeting ASO 4 weeks post-dosing. However, in this study we measured ASO concentration and activity only in xenografted microglia and are not able to compare ASO effect between different cell types. Thus, further studies will be necessary to decipher the adequate ASO dosing to allow long-term silencing, beyond 4-weeks tested here, in microglia. This will allow us to study chronic effects of ASO-mediated microglia manipulation under pathological conditions in disease-relevant models.

Our data in the xenografted animals also indicate that the ASOs are pharmacologically active in the CNS but there is also exposure in peripheral organs such as liver and kidney. This is consistent with the reported biodistribution of ASOs after direct CSF delivery of ASOs in rodents [34, 35]. Although it was previously shown that ASO levels in the plasma after direct CNS delivery are multiple orders of magnitude lower compared to ASO levels after peripheral administration [34], we cannot confirm the potentially lower targeting of *APOE* and *TREM2* in peripheral organs, as these are not expressed in the systemic compartment of our xenotransplantation.

Several studies demonstrated that the absence of mouse Trem2 aggravates Aβ and Tau pathology, promotes neuritic dystrophy and facilitates Tau seeding in mouse models of AD [98, 99, 101–103]. Studies examining chronic Trem2 activation in AD mouse models, have shown lowered Aβ pathology after anti-Trem2 antibody treatment [104–107], while effects on Tau pathology remain conflicting [105, 106]. TREM2 function may depend on other factors such as age, insult/injury type and the pre-existence of pathology [97, 108–111]. Acute reduction of mouse *Trem2* in a mouse model of AD in late stages of the phenotype increased microglia activation and phagocytic activity and reduced pathological protein accumulation [47]. These data suggest that acute ASO-mediated rather than constitutive genetic *TREM2* lowering strategies may be needed to study the role of TREM2 in microglial biology in disease. These age-dependent effects were also observed when reducing ApoE levels in mouse models of β-amyloidosis [45]. Specifically, lowering ApoE levels prior to pathology initiation ameliorated the phenotype whereas ASO-mediated reduction of ApoE modulated Aβ plaque accumulation [45]. Importantly, unlike Trem2, ApoE is expressed in multiple cell types in the brain and expressed in microglia only under pathological conditions. Recent study demonstrated selective knockout of ApoE in microglia does not affect plaque formation nor their transition into a DAM state [112], suggesting the role of ApoE in AD is dependent on multiple cell types, and not driven by microglia. In the same line, we have previously shown that the genetic manipulation of APOE in xenotransplanted microglia have only mild effects on their response to amyloid-β pathology [22]. Here, we reduced APOE expression selectively in microglia, and observed downregulation of DAM associated genes. However, further studies will be necessary to evaluate how selective knockdown of ApoE in microglia would affect amyloid pathology. Furthermore, it is important to further evaluate the contribution of the other APOE expressing cells in this complex multi-cellular contribution system of APOE. Collectively, these data demonstrate that the time of ASO-mediated target expression modulation is essential for the therapeutically relevant outcome and highlight the importance of evaluating ASO effects in animal models of disease.

## Conclusions

Our data reveal for the first time that *APOE* and *TREM2* expression levels can be reduced with ASOs in human microglia in vitro and in vivo, and that leads to alterations in the phenotypic response of microglia to amyloid-β pathology. Our work highlights the potential of ASOs as a research tool to study the functions of APOE and TREM2 in neuroinflammation and as a potential therapeutic modality to interfere with the role of microglia and neuroinflammation in neurodegenerative diseases.

### Supplementary Information

**Supplementary Material 1.**

**Supplementary Material 2.**

**Supplementary Material 3.**

**Supplementary Material 4.**

**Supplementary Material 5. **

**Supplementary Material 6. **

**Supplementary Material 7. **

**Supplementary Material 8. **

**Supplementary Material 9. **

**Supplementary Material 10. **

**Supplementary Material 11. **

**Additional file 12 Supplemental Fig. 1. **ASO screening cascade and primer design.

**Additional file 13 Supplemental Fig. 2. **Gating strategy for sorting xenotransplanted human and endogenous mouse microglia employing flow cytometry.

**Additional file 14 Supplemental Fig. 3. **APOE and TREM2 ASO screening in THP-1 cells.

**Additional file 15 Supplemental Fig. 4. **Microglial differentiation of iPSCs and target expression in cultured microglia.

**Additional file 16 Supplemental Fig. 5. **ASOs are internalized and pharmacologically active in cultured human microglia.

**Additional file 17 Supplemental Fig. 6. **Fast ASO internalization dynamics in cultured human microglia.

**Additional file 18 Supplemental Fig. 7. **Cellular and secreted APOE protein levels in cultured human isogenic gene-edited APOE microglia.

**Additional file 19 Supplemental Fig. 8. **ASOs are internalized and pharmacologically active in H9 hESC-derived microglia.

**Additional file 20 Supplemental Fig. 9. **ASO is widely distributed in the brain 7 days after icv ASO administration.

**Additional file 21 Supplemental Fig. 10. **Human APOE and TREM2 are expressed at the RNA level in human xenografted microglia in mouse brain.

**Additional file 22 Supplemental Fig. 11. **Microglial response to amyloid-β fibrils upon ASO-mediated APOE and TREM2 knockdown.

**Additional file 23 Supplemental Fig. 12. **Enrichment of microglial subtype markers in response to treatment**.**

**Additional file 24 Supplemental Fig. 13. **Early safety assessment of lead APOE and TREM2 ASOs**. **

**Additional file 25 Supplemental Fig. 14. **Immunogenicity of lead APOE ASO in human cultured microglia and human whole blood.

**Additional file 26 Supplemental Fig. 15**. Immunogenicity of lead TREM2 ASO in human cultured microglia.

## Data Availability

The RNAseq data from human microglia in vivo can be accessed via GEO (ID: GSE219284). The data from the microarray to determine off target effects can be accessed via GSE243243.
