# Supplementary material for: Regulation of human microglial gene expression and function via RNAase-H active antisense oligonucleotides in vivo in Alzheimer’s disease
Source: Mol Neurodegener. 2024 Apr 24;19:37. doi: 10.1186/s13024-024-00725-9 (PMC11040766; doi:10.1186/s13024-024-00725-9)
Supplement: Supplementary file 12 — Additional file 12 Supplemental Fig. 1. ASO screening cascade and primer design. [file 13024_2024_725_MOESM12_ESM.pdf]

A

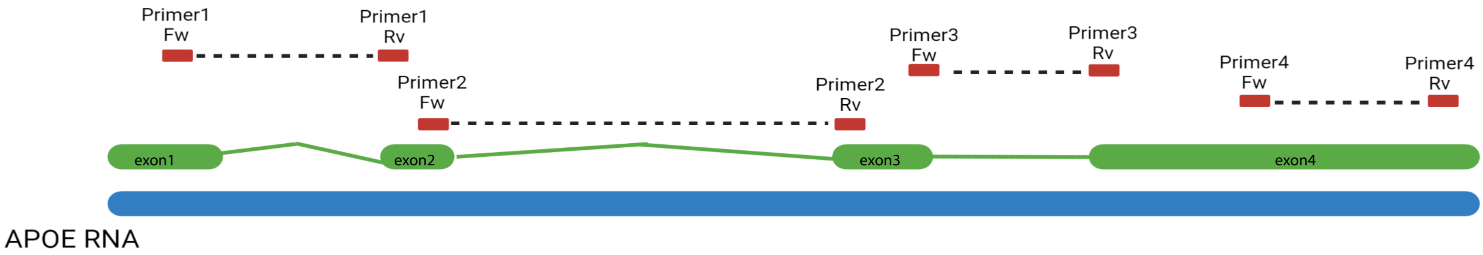

B

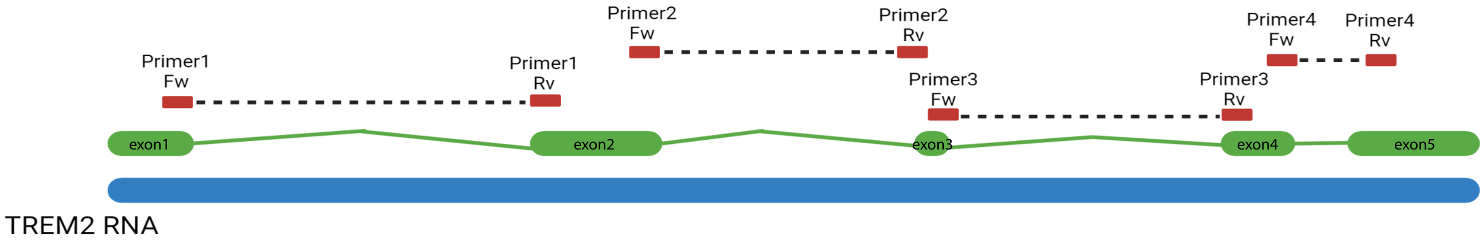

C

Cell line type	Acute monocytic leukemia		Neuroblastoma			Colorectal carcinoma		Melanoma	Breast Carcinoma
	THP-1	K-562	SH-SY5Y	SK-N-MC	Kelly	HCT	Caco2	Sk-Mel1	T-46D
Assay/ CT value									
Hs_APOE-2	17.0	20.476	28.5	22.7	25.3	29.2	33.055	19.388	21.310
Hs_APOE-4	18.6	21.393	29.9	25.2	26.3	30.8	25.957	19.388	21.933
Hs_TREM2-2	20.7	31.251	Undetermined	Undetermined	Undetermined	Undetermined	29.110	32.588	32.505
Hs_TREM2-4	20.4	29.029	27.8	34.7	33.2	29.3	25.652	28.910	30.007
