# Supplementary figures and images for: Regulation of human microglial gene expression and function via RNAase-H active antisense oligonucleotides in vivo in Alzheimer’s disease
Source: Mol Neurodegener. 2024 Apr 24;19:37. doi: 10.1186/s13024-024-00725-9 (PMC11040766; doi:10.1186/s13024-024-00725-9)

A

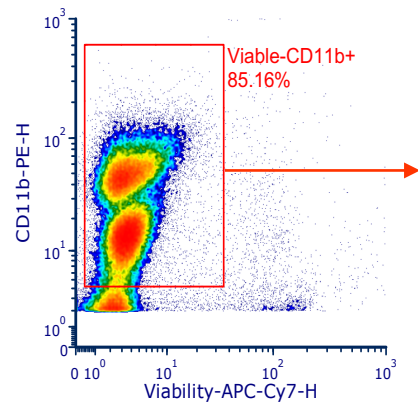

B

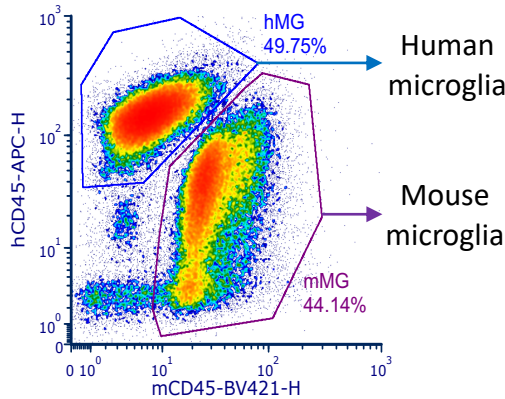

Supplement: Supplementary file 13 — Additional file 13 Supplemental Fig. 2. Gating strategy for sorting xenotransplanted human and endogenous mouse microglia employing flow cytometry. [file 13024_2024_725_MOESM13_ESM.pdf]

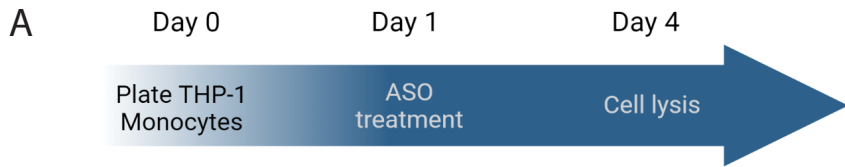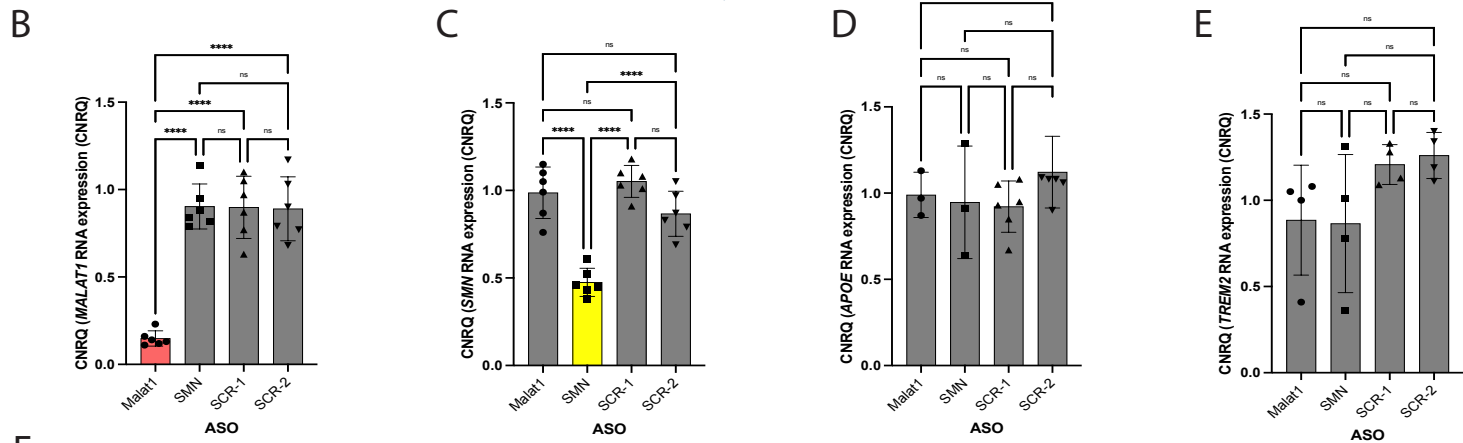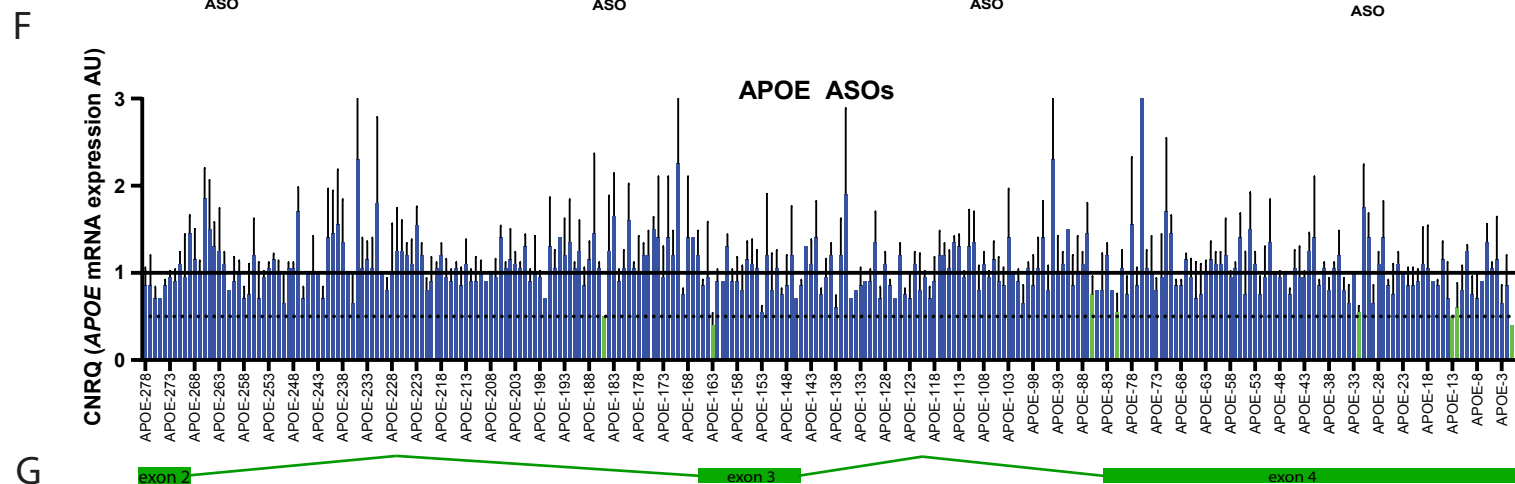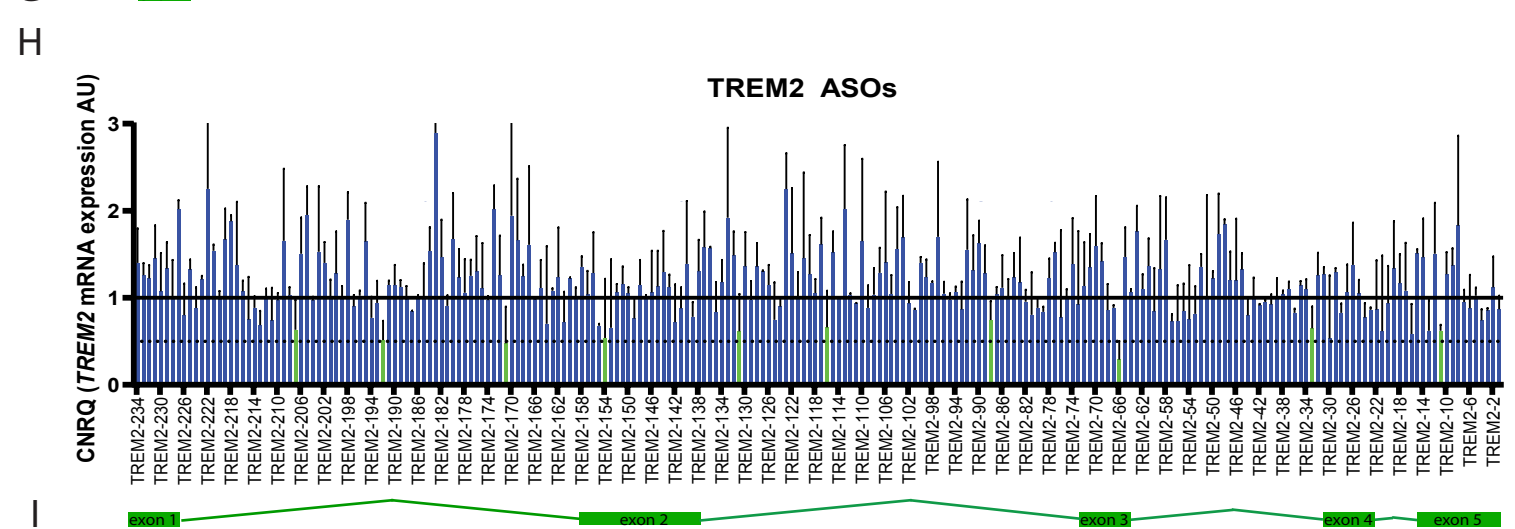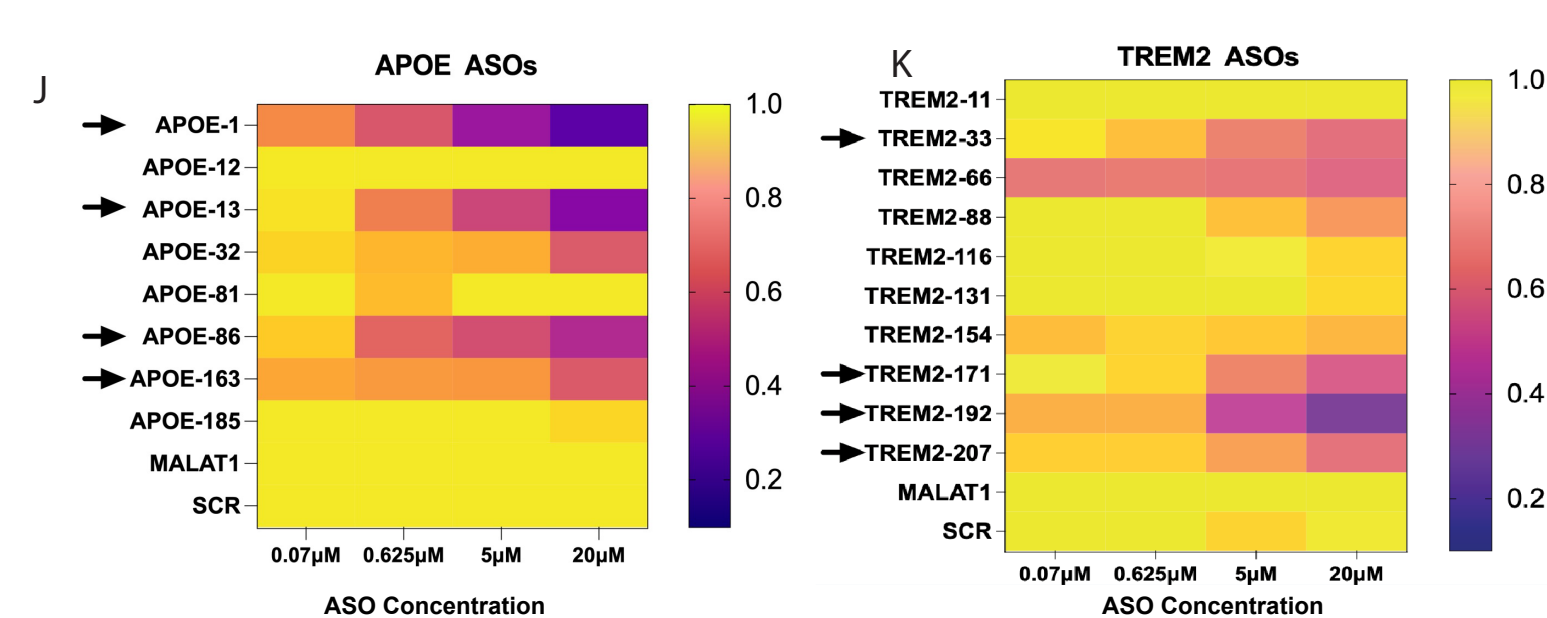

Supplement: Supplementary file 14 — Additional file 14 Supplemental Fig. 3. APOE and TREM2 ASO screening in THP-1 cells. [file 13024_2024_725_MOESM14_ESM.pdf]

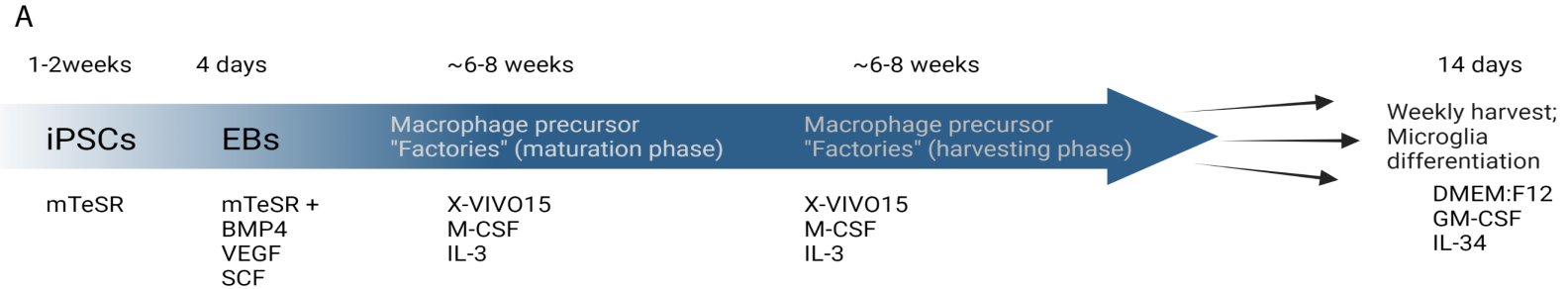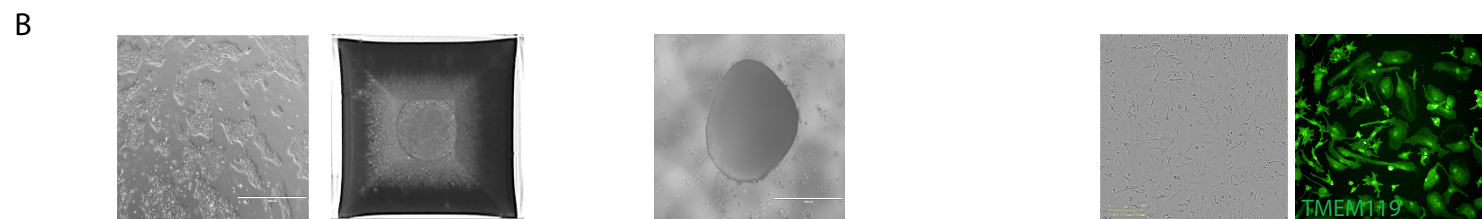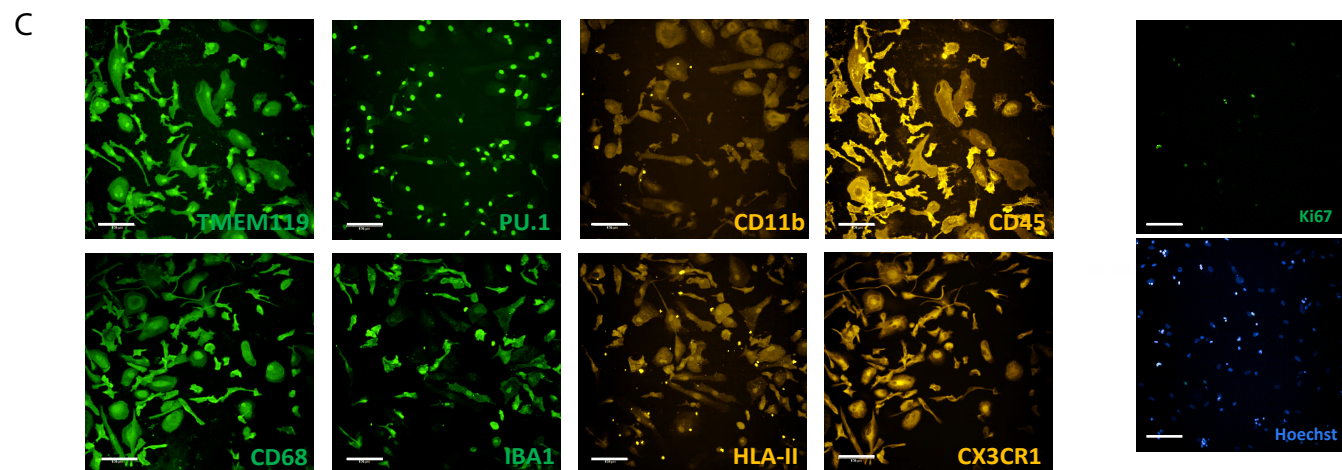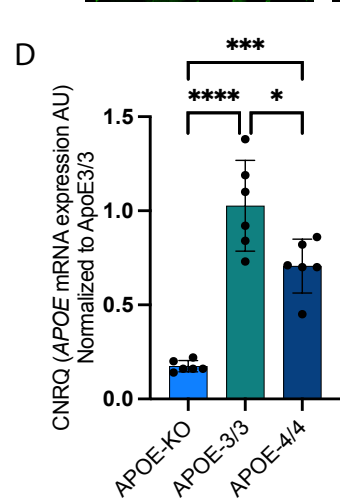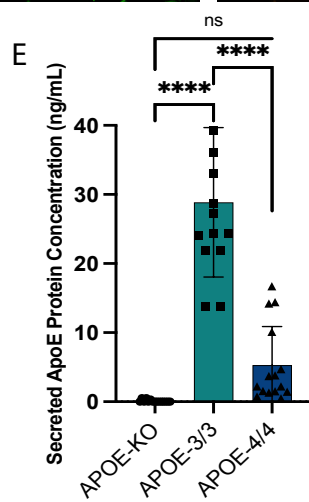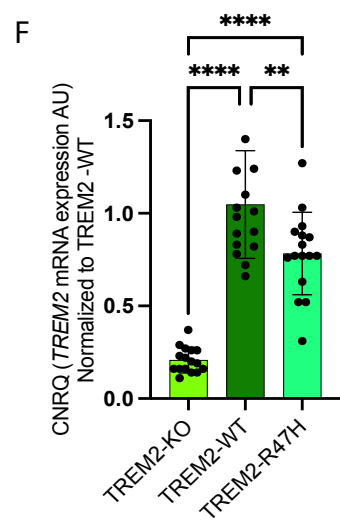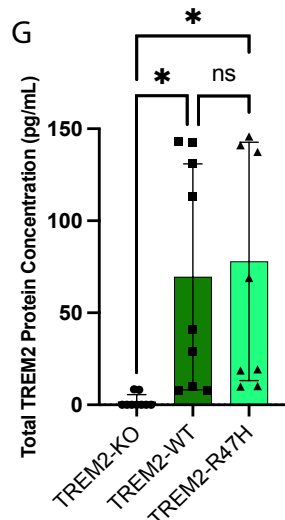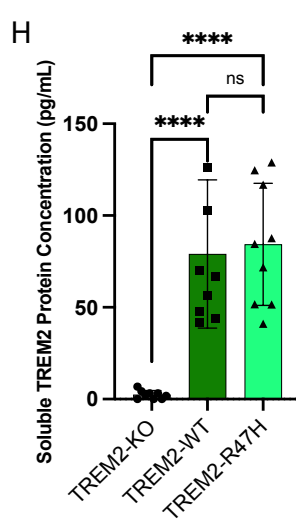

Supplement: Supplementary file 15 — Additional file 15 Supplemental Fig. 4. Microglial differentiation of iPSCs and target expression in cultured microglia. [file 13024_2024_725_MOESM15_ESM.pdf]

A

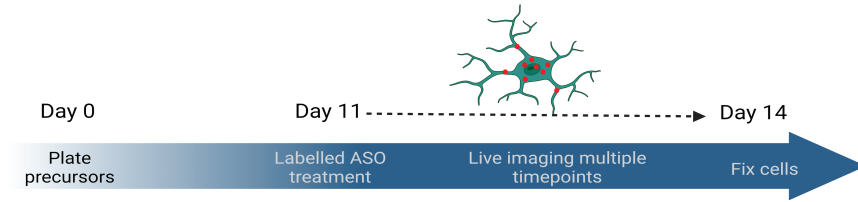

B

UKBi011-A3

BIONi010-C

SIGi001-A

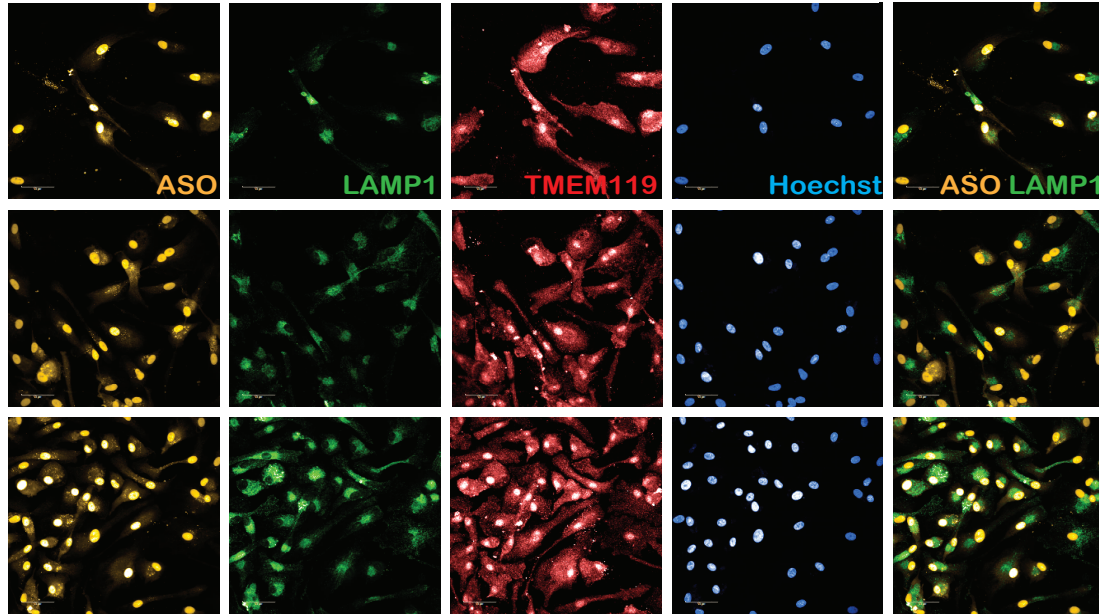

C

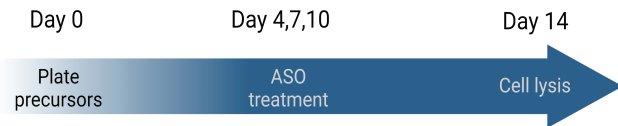

D

UKBi011-A-3  
APOE<sup>ε3/ε3</sup>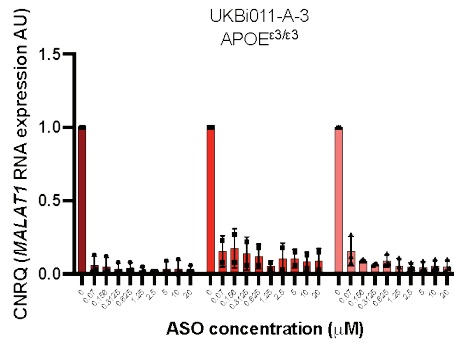

E

UKBi011-A  
APOE<sup>ε4/ε4</sup>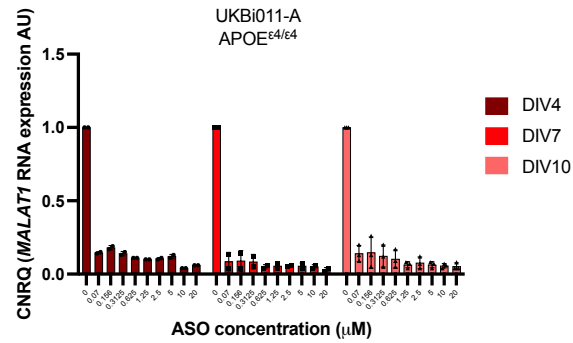

Supplement: Supplementary file 16 — Additional file 16 Supplemental Fig. 5. ASOs are internalized and pharmacologically active in cultured human microglia. [file 13024_2024_725_MOESM16_ESM.pdf]

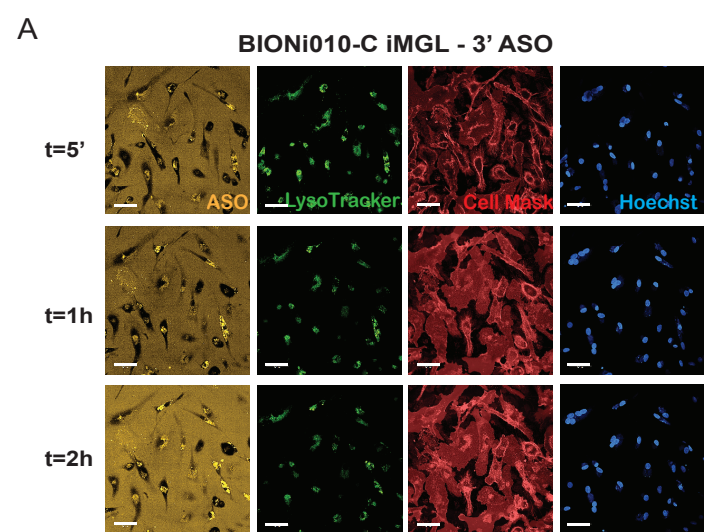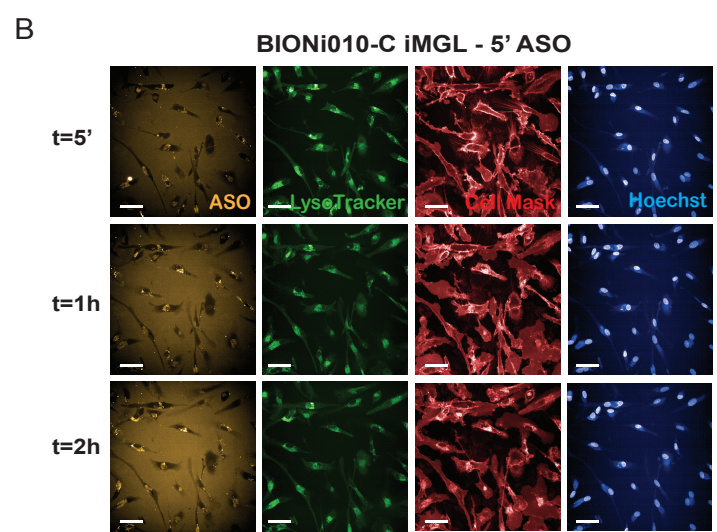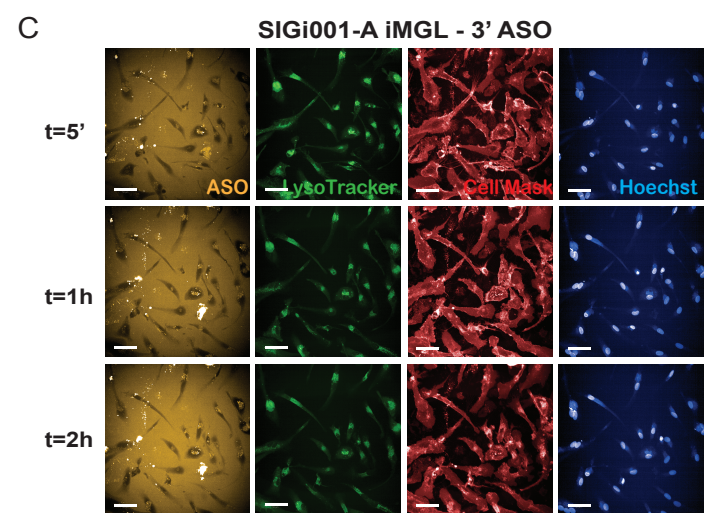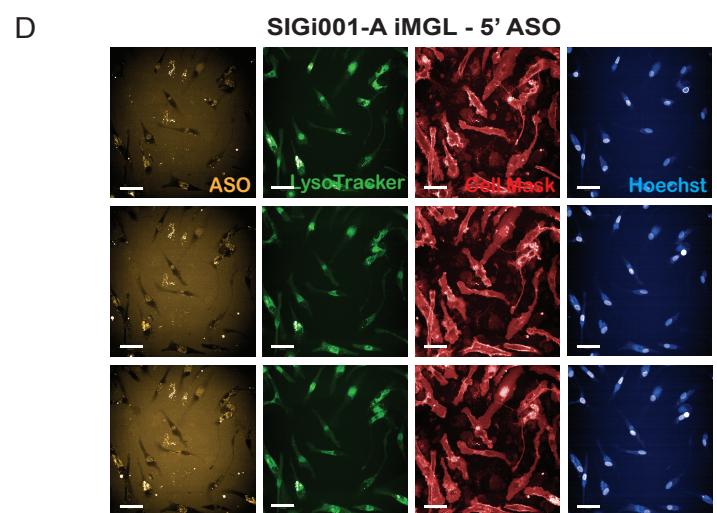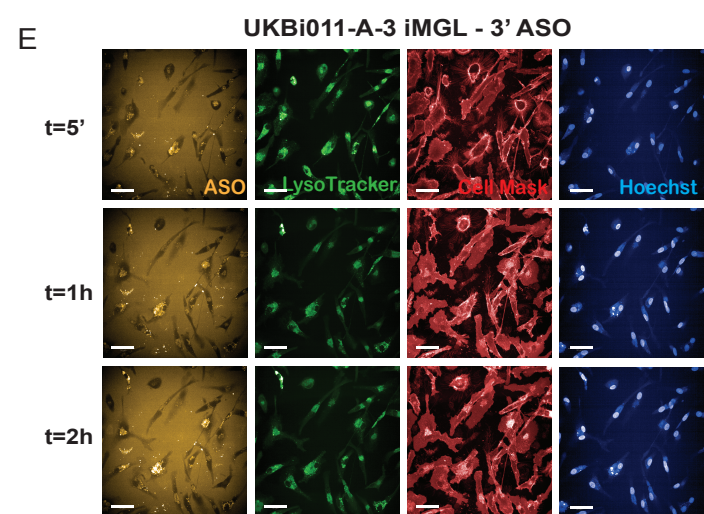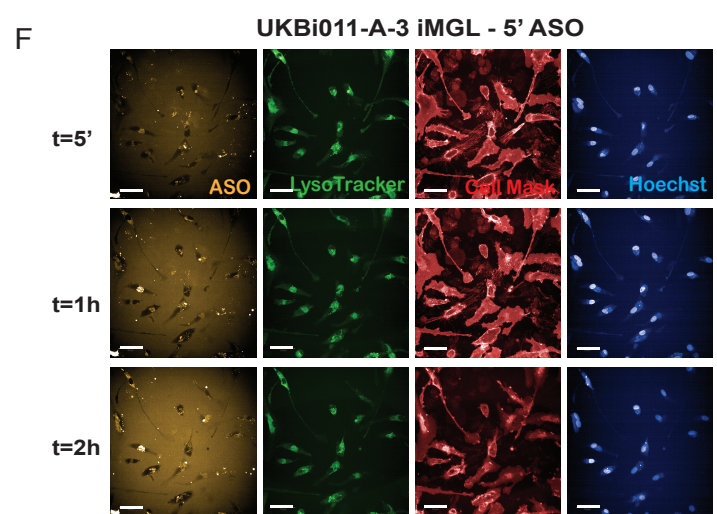

Supplement: Supplementary file 17 — Additional file 17 Supplemental Fig. 6. Fast ASO internalization dynamics in cultured human microglia. [file 13024_2024_725_MOESM17_ESM.pdf]

A

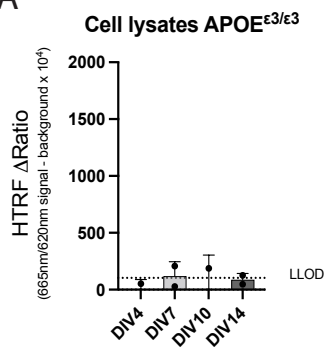

B

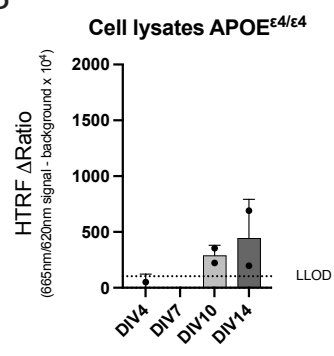

C

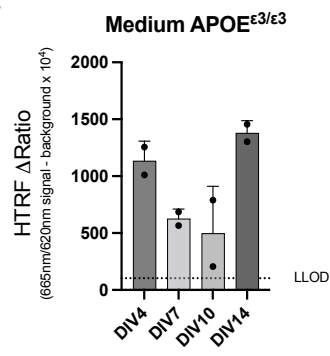

D

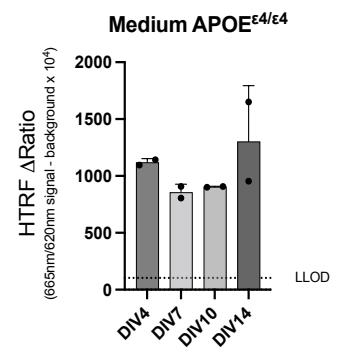

Supplement: Supplementary file 18 — Additional file 18 Supplemental Fig. 7. Cellular and secreted APOE protein levels in cultured human isogenic gene-edited APOE microglia. [file 13024_2024_725_MOESM18_ESM.pdf]

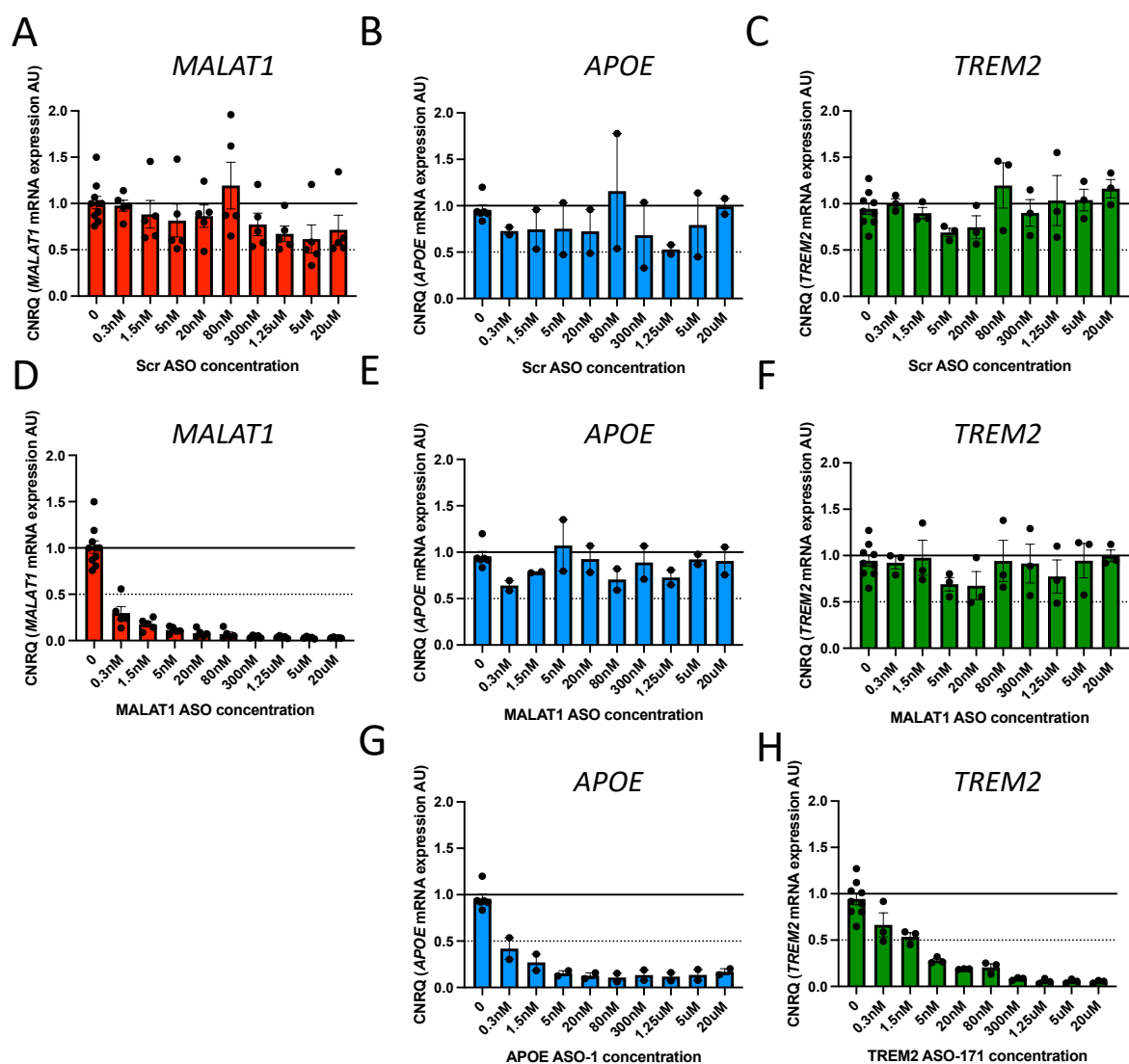

Supplement: Supplementary file 19 — Additional file 19 Supplemental Fig. 8. ASOs are internalized and pharmacologically active in H9 hESC-derived microglia. [file 13024_2024_725_MOESM19_ESM.pdf]

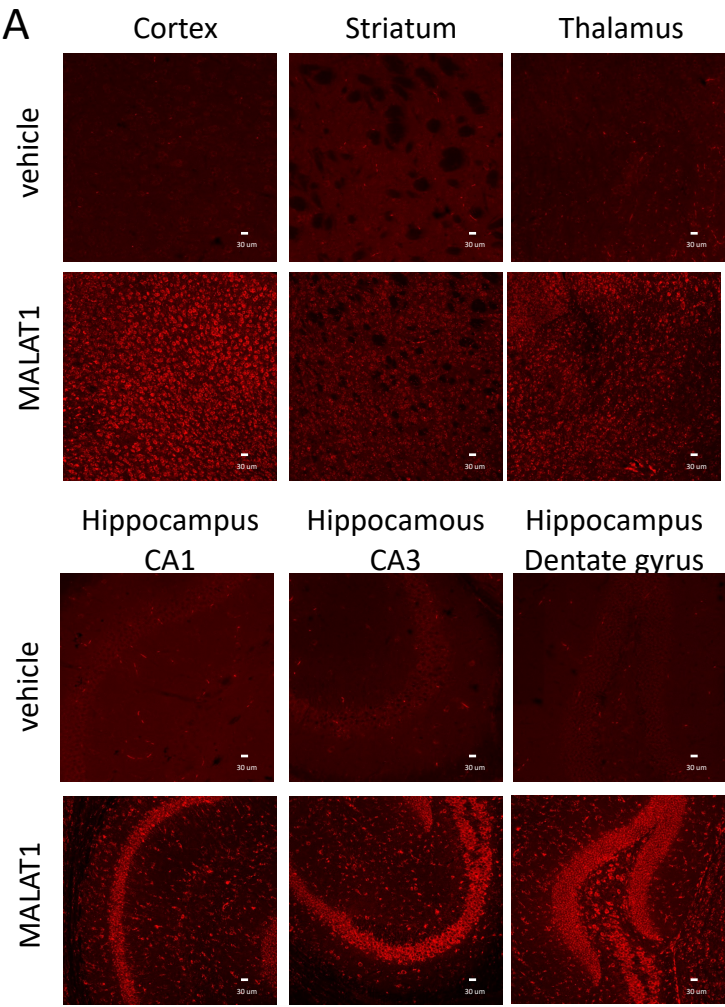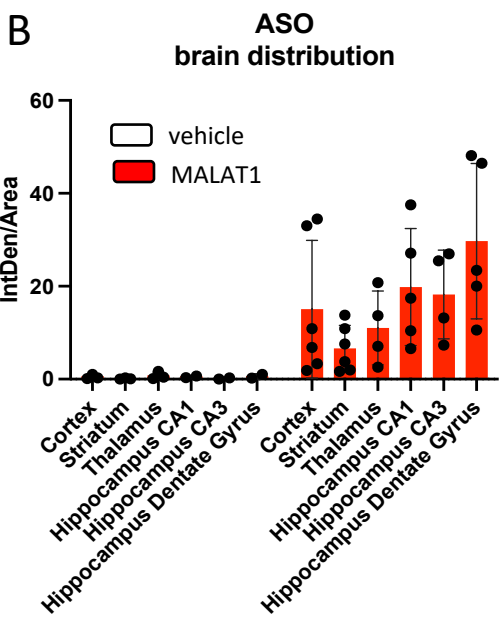

Supplement: Supplementary file 20 — Additional file 20 Supplemental Fig. 9. ASO is widely distributed in the brain 7 days after icv ASO administration. [file 13024_2024_725_MOESM20_ESM.pdf]

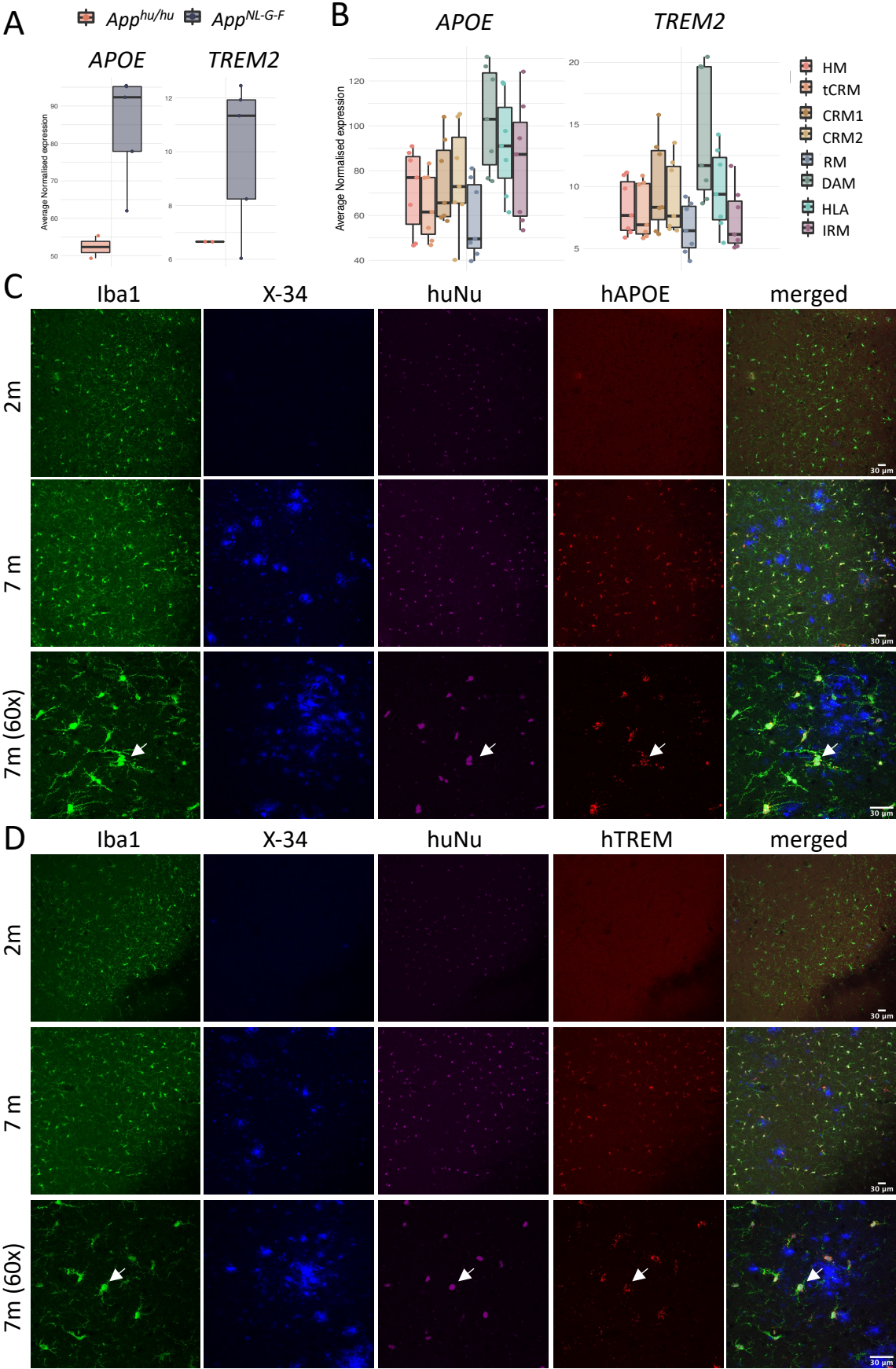

Supplement: Supplementary file 21 — Additional file 21 Supplemental Fig. 10. Human APOE and TREM2 are expressed at the RNA level in human xenografted microglia in mouse brain. [file 13024_2024_725_MOESM21_ESM.pdf]

**A**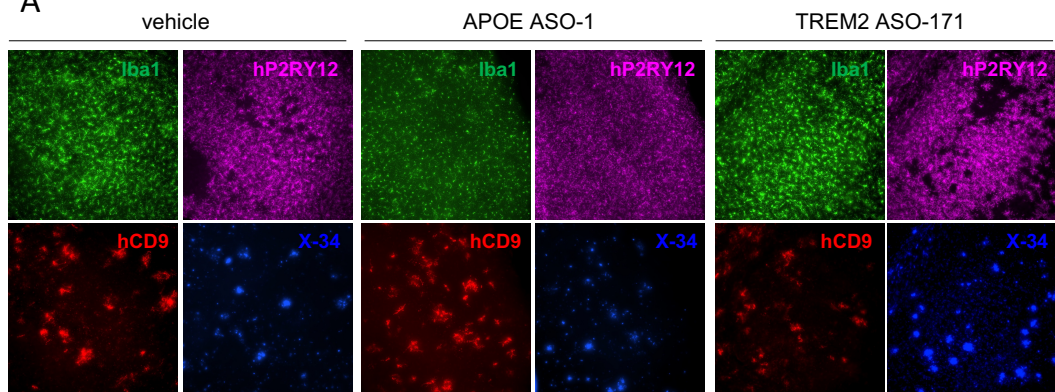**B**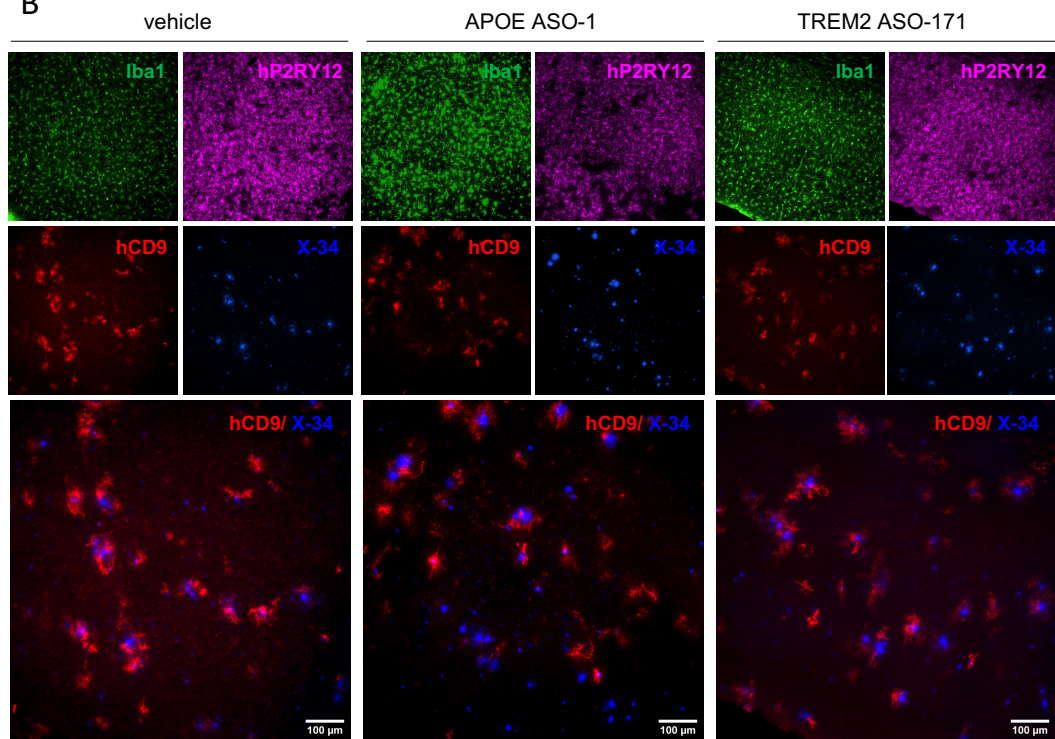**C**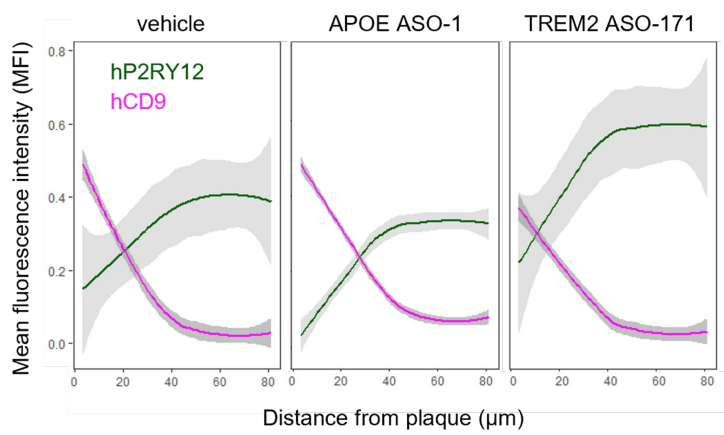**D**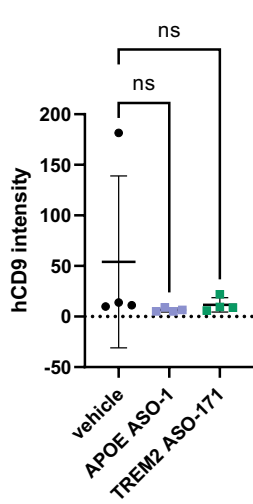

Supplement: Supplementary file 22 — Additional file 22 Supplemental Fig. 11. Microglial response to amyloid-β fibrils upon ASO-mediated APOE and TREM2 knockdown. [file 13024_2024_725_MOESM22_ESM.pdf]

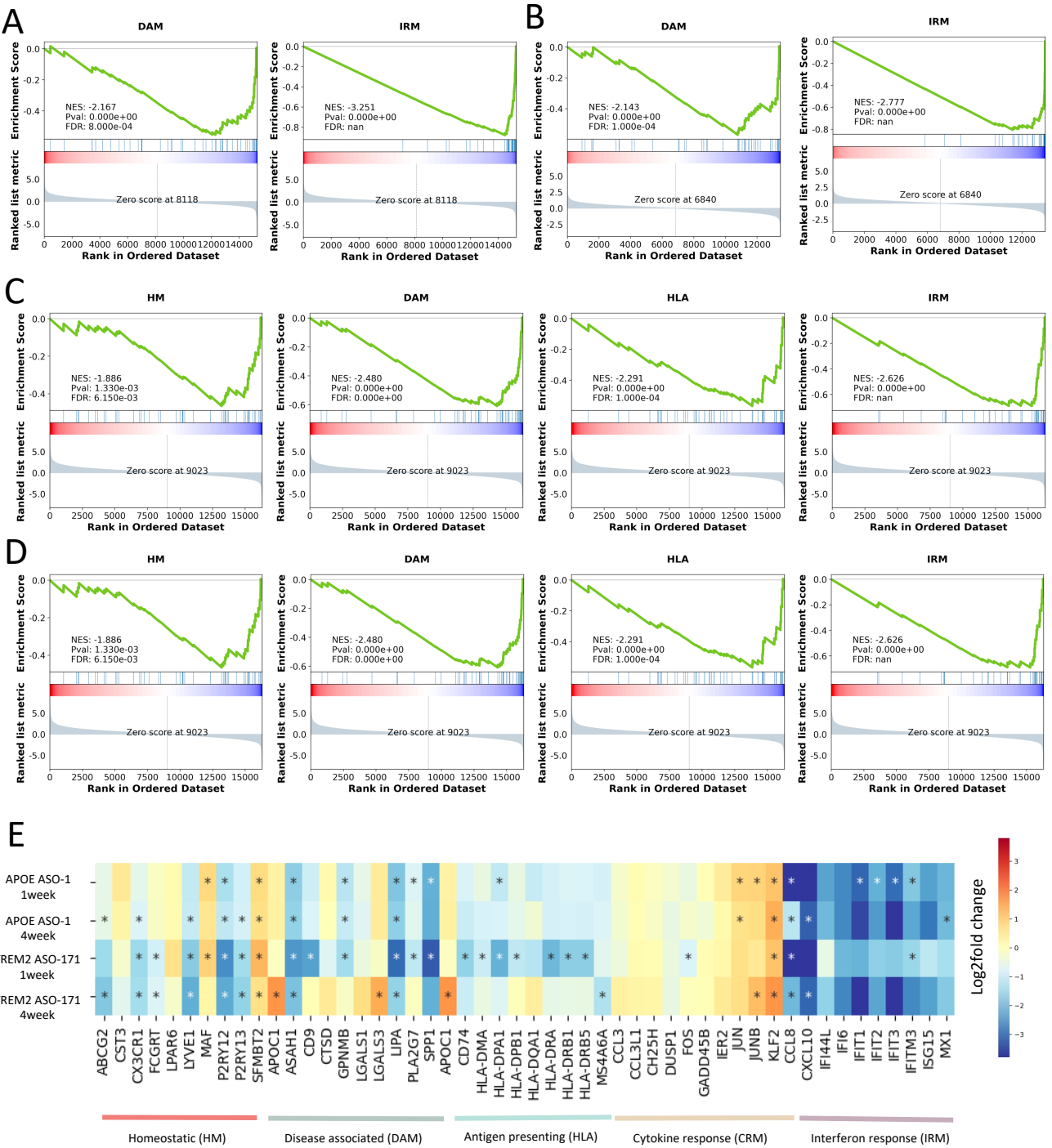

Supplement: Supplementary file 23 — Additional file 23 Supplemental Fig. 12. Enrichment of microglial subtype markers in response to treatment. [file 13024_2024_725_MOESM23_ESM.pdf]

A

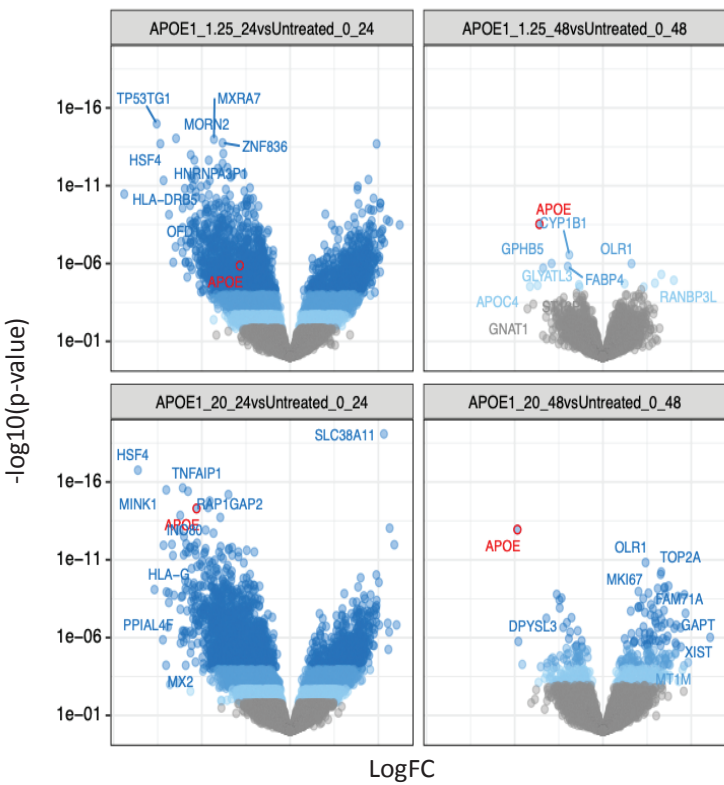

B

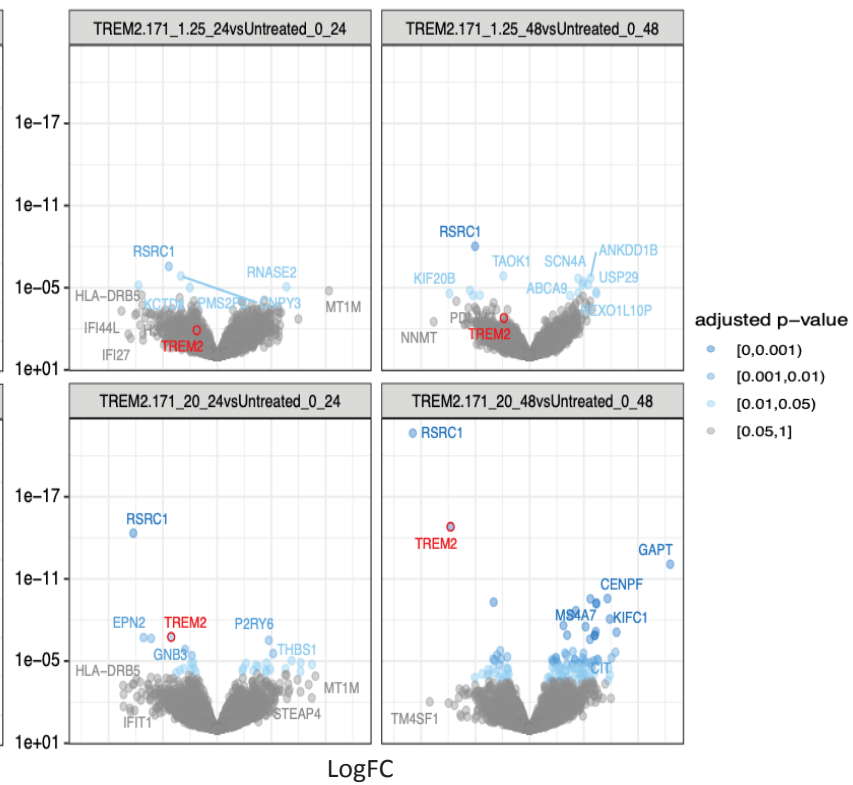

C

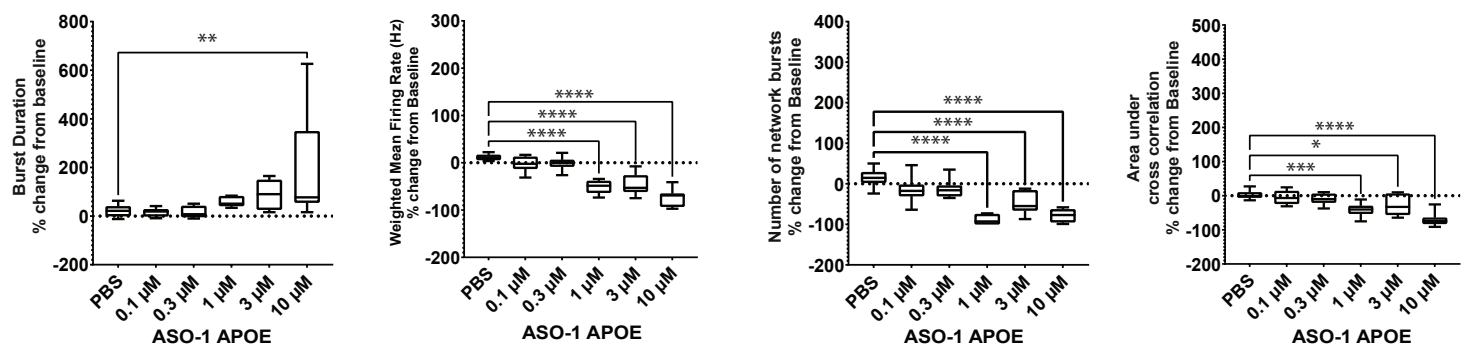

D

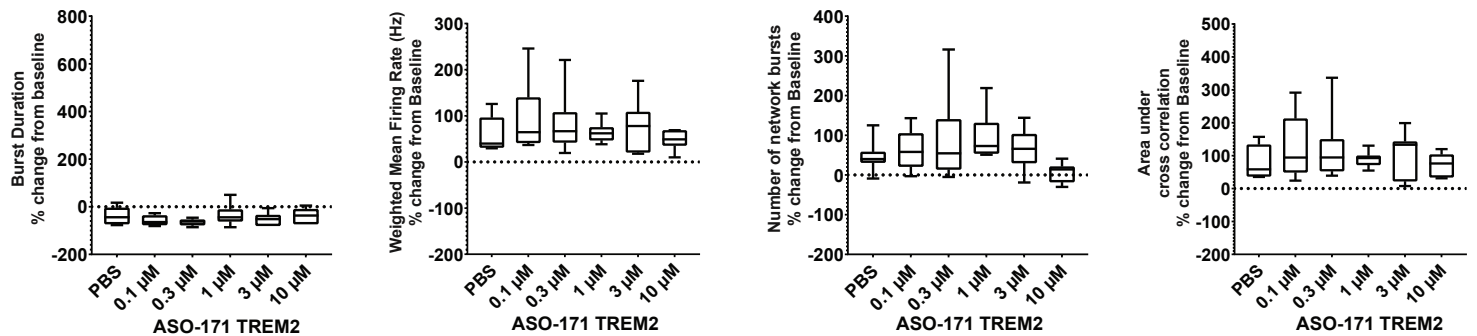

Supplement: Supplementary file 24 — Additional file 24 Supplemental Fig. 13. Early safety assessment of lead APOE and TREM2 ASOs. [file 13024_2024_725_MOESM24_ESM.pdf]

A

APOE<sup>ε3/ε3</sup>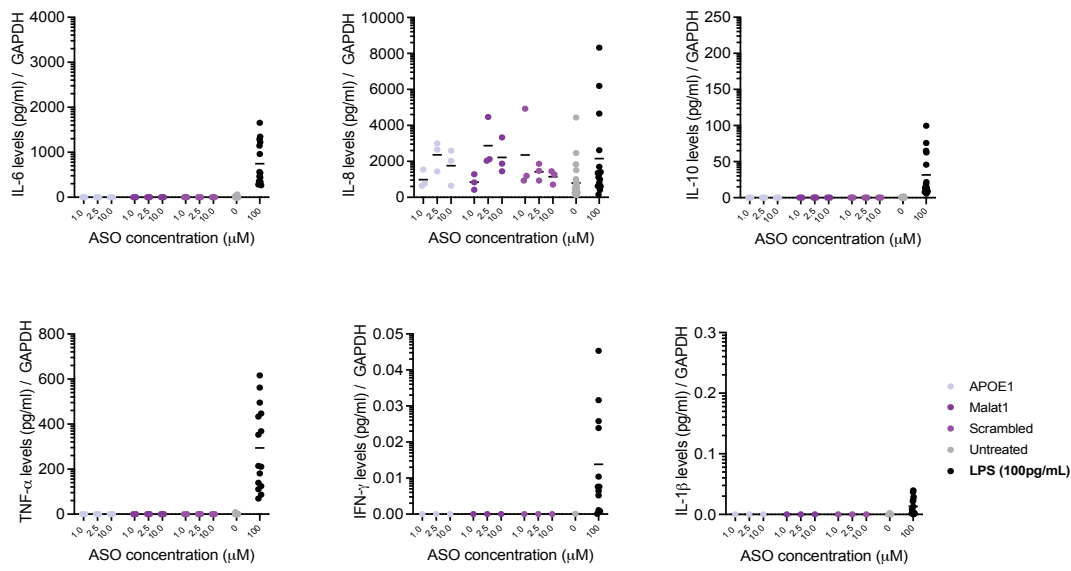

B

APOE<sup>ε4/ε4</sup>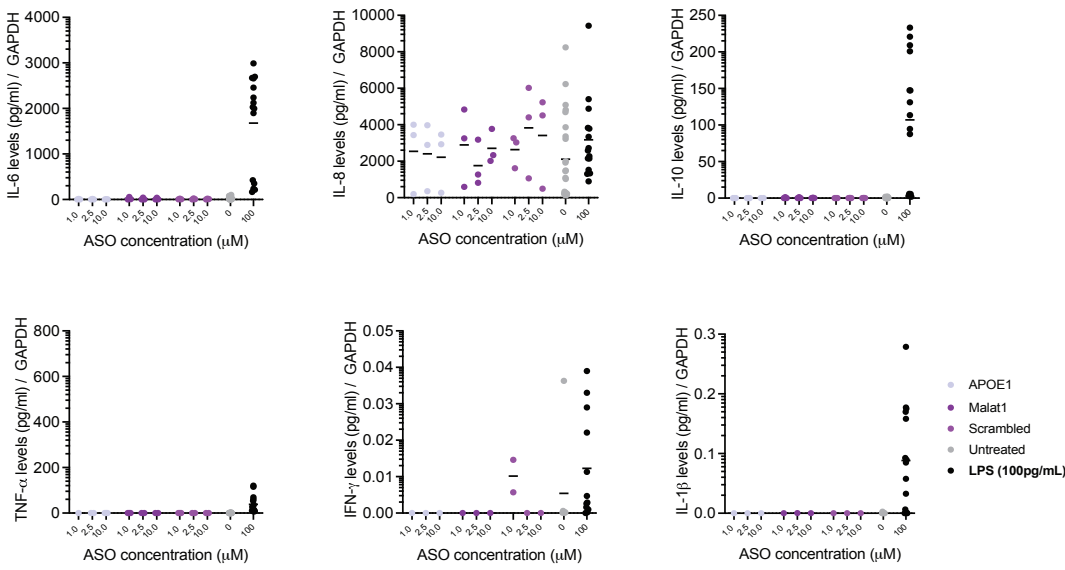

C

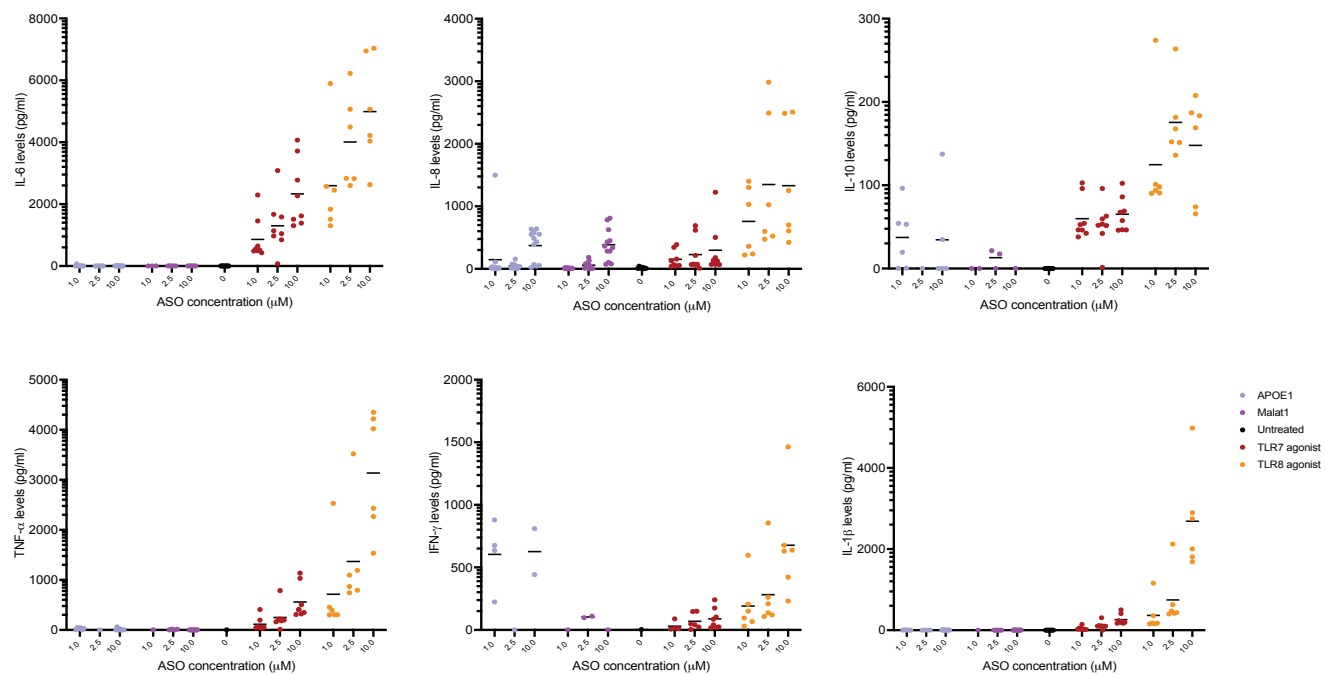

Supplement: Supplementary file 25 — Additional file 25 Supplemental Fig. 14. Immunogenicity of lead APOE ASO in human cultured microglia and human whole blood. [file 13024_2024_725_MOESM25_ESM.pdf]

A

TREM2<sup>WT/WT</sup>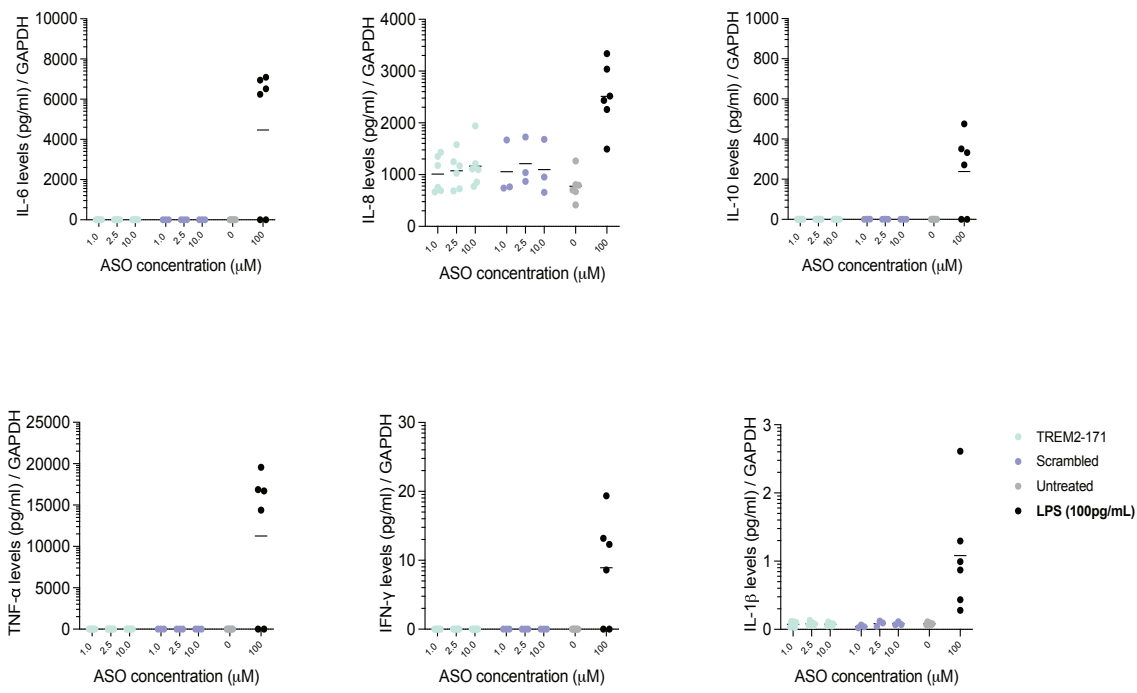

B

TREM2<sup>R47H/R47H</sup>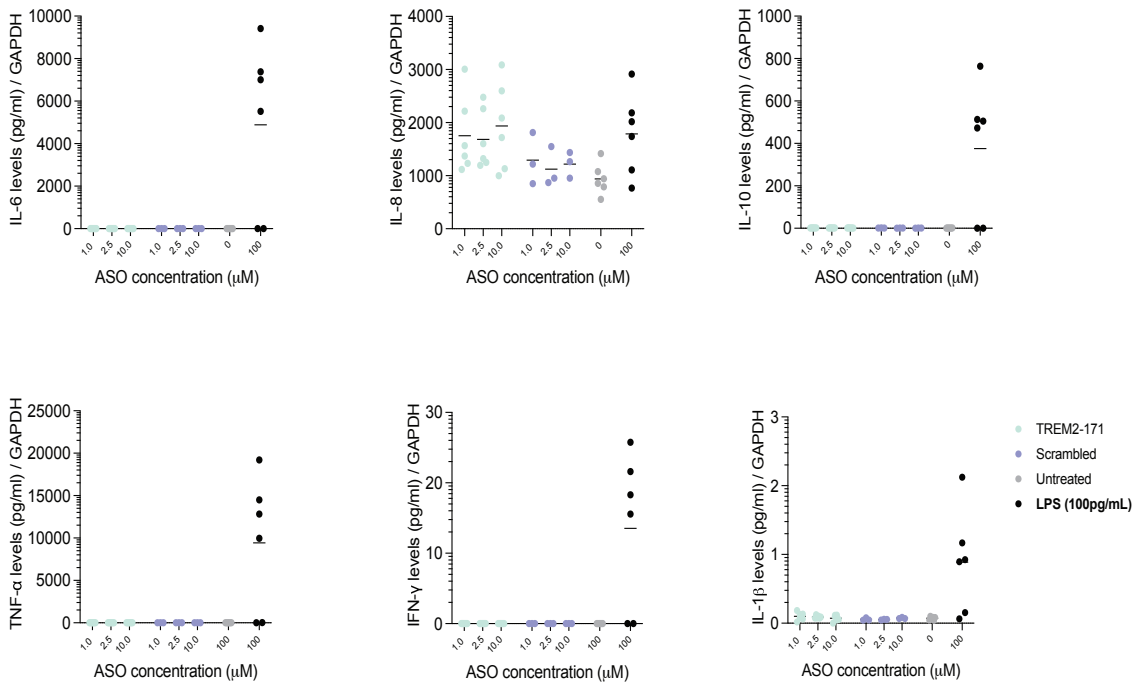

Supplement: Supplementary file 26 — Additional file 26 Supplemental Fig. 15. Immunogenicity of lead TREM2 ASO in human cultured microglia. [file 13024_2024_725_MOESM26_ESM.pdf]
